# Mosquito-Textile Physics: A Mathematical Roadmap to Insecticide-Free, Bite-Proof Clothing for Everyday Life

**DOI:** 10.3390/insects12070636

**Published:** 2021-07-13

**Authors:** Kun Luan, Andre J. West, Marian G. McCord, Emiel A. DenHartog, Quan Shi, Isa Bettermann, Jiayin Li, Nicholas V. Travanty, Robert D. Mitchell, Grayson L. Cave, John B. Strider, Yongxin Wang, Florian Neumann, Tobias Beck, Charles S. Apperson, R. Michael Roe

**Affiliations:** 1 Department of Forest Biomaterials, College of Natural Resources, NC State University, Raleigh, NC 27695, USA; kluan@ncsu.edu (K.L.); marianmccord@gmail.com (M.G.M.); 2 Department of Textile Engineering, Chemistry and Science, Wilson College of Textiles, NC State University, Raleigh, NC 27695, USA; eadenhar@ncsu.edu (E.A.D.); quan.shi@hotmail.com (Q.S.); ywang45@ncsu.edu (Y.W.); 3 Department of Textile and Apparel, Technology and Management, Wilson College of Textiles, NC State University, Raleigh, NC 27695, USA; jli54@ncsu.edu; 4 Institut für Textiltechnik der RWTH, Aachen University, 52062 Aachen, Germany; isa.bettermann@ita.rwth-aachen.de (I.B.); Florian.Neumann@delcotex.de (F.N.); tobias.beck@kob.de (T.B.); 5 Department of Entomology & Plant Pathology, College of Agriculture and Life Sciences, NC State University, Raleigh, NC 27695, USA; travanty@gmail.com (N.V.T.); Mitchell.Robert@epa.gov (R.D.M.III); glcave@ncsu.edu (G.L.C.); JSTRIDER1@nc.rr.com (J.B.S.); apperson@ncsu.edu (C.S.A.); 6 Comparative Medicine Institute, NC State University, Raleigh, NC 27695, USA

**Keywords:** mosquito, bite-proof garment, model, textile, non-insecticidal, physical barrier

## Abstract

**Simple Summary:**

Mosquitoes can bite across clothing and transmit disease. This is prevented with pesticides applied to clothing. We developed non-insecticidal cloth and garments that provided 100% protection, were comfortable and look-like and feel-like regular clothing.

**Abstract:**

Garments treated with chemical insecticides are commonly used to prevent mosquito bites. Resistance to insecticides, however, is threatening the efficacy of this technology, and people are increasingly concerned about the potential health impacts of wearing insecticide-treated clothing. Here, we report a mathematical model for fabric barriers that resist bites from *Aedes aegypti* mosquitoes based on textile physical structure and no insecticides. The model was derived from mosquito morphometrics and analysis of mosquito biting behavior. Woven filter fabrics, precision polypropylene plates, and knitted fabrics were used for model validation. Then, based on the model predictions, prototype knitted textiles and garments were developed that prevented mosquito biting, and comfort testing showed the garments to possess superior thermophysiological properties. Our fabrics provided a three-times greater bite resistance than the insecticide-treated cloth. Our predictive model can be used to develop additional textiles in the future for garments that are highly bite resistant to mosquitoes.

## 1. Introduction

Mosquito-transmitted diseases are a major, global human health problem [1]. Pathogens transmitted by mosquito bites cause illnesses that kill an estimated 700,000 people each year [2]. Personal protection from mosquito-borne diseases has largely involved the use of chemical repellents applied to clothing and skin or insecticides either sprayed on garments before use or bound to textiles or garments to survive multiple uses and washes. Insecticide-treated textiles in the form of long-lasting insecticidal bed nets (LLINs) are also used for mosquito control in malaria-endemic areas. According to the World Health Organization, pyrethroid-treated bed nets have played a vital role in reducing malaria in Africa (World Health Organization (2019), World Malaria Report, WHO, Geneva, Switzerland [3]). Between 2000 and 2015, an estimated 663 million clinical cases of malaria were averted, of which 68% were attributed to the wide-area deployment of LLINs [4]. The use of insecticide-treated curtains [5,6], long-lasting insecticidal bed nets, and insecticide-treated clothing [7] have substantially reduced the transmission of vector-borne pathogens. Unfortunately, the widespread use of insecticides has also led to the development of insecticide-resistant mosquitoes, and the insecticides are now ineffective in many places [8].

Furthermore, in spite of the benefits from insecticide-treated textiles, there are potential deleterious health effects [7]. Since the garments are in continuous contact with the skin, the potential for insecticide exposure is increased. Permethrin is the principal insecticide used to treat clothing [9]. Development of safe, alternative insecticides for textiles is costly and requires regulatory approvals for new chemistry. Because of the potential health risks from the use of pesticides, people today given a choice prefer to avoid insecticide exposure. Development of mosquito-bite-resistant garments without insecticides that are comfortable and as effective (or more effective) than insecticide-treated garments would be a “game changer” and provide to the public, for the first time, a choice. We have achieved this objective.

Fabrics inherently are favorable structures for producing physical barriers against insects. Textiles have a three-dimensional structure assembled with interlacements or intermeshing fibers and yarns in organized patterns [10]. The design of fibers and yarns produce textile structures with a diverse range of properties, some of which could provide insect protection [11]. Fabrics have been specifically designed as physical barriers against environmental factors such as water [12], airflow [13], or heat and cold [14]. The existence of open spaces between fibers and yarns ensures fabric breathability and thermal comfort [15]; however, these spaces produce pores through a fabric allowing penetration of human olfactory (smell) and thermal (temperature) cues that attract mosquitoes [16]. The fabric pores serve as channels for the mosquito to take a blood meal. The objective of our research is to develop a mathematical model to predict blood feeding across textiles that could be used to develop a practical, non-insecticidal, bite-resistant garment.

## 2. Materials and Methods

### 2.1. Mosquitoes

**Figure 1:**
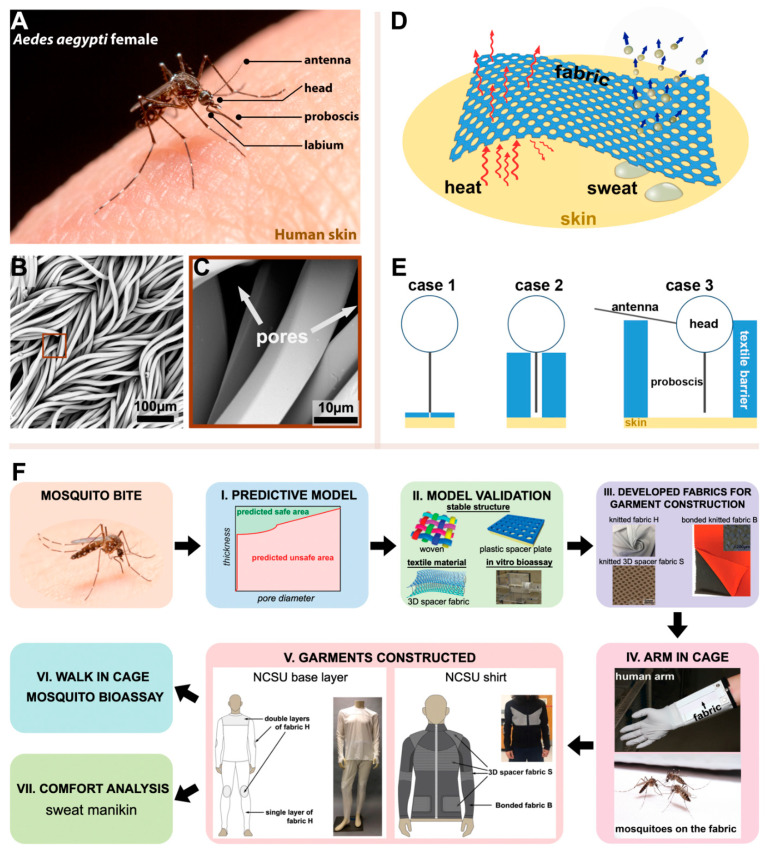
Principle of a bite-resistant textile structure against *Aedes aegypti*. (**A**) An *Ae. aegypti* adult female feeding on the blood beneath human skin. (**B**) SEM image of a knit structure. (**C**) Example of pores formed by the filaments in the knit structure. (**D**) Heat and moisture management of a fabric. (**E**) The proposed three cases for mosquito-bite resistance. (**F**) Research steps for the design of bite-resistant garments.

Adult, female yellow fever mosquitoes, *Aedes aegypti* (Diptera: Culicidae), are a major vector of pathogens that cause animal and human diseases worldwide [17,18,19] and were used as a model insect for the studies that follow. *Ae. aegypti* females (Figure 1A and Figure S1) were obtained from a colony maintained in the Dearstyne Entomology Laboratory at North Carolina State University, Raleigh, NC, USA. The mosquito colony has been continuously reared for approximately 5 years and is free of pathogens. Adults were kept at 27 °C and 80% relative humidity with a 14:10 h light: dark photoperiod. Adults were provisioned with a 10% sucrose solution (in distilled water) *ad libitum*. To obtain eggs for colony maintenance, female mosquitoes were fed porcine blood (obtained from a local abattoir) using an *in vitro* blood-feeding device (described later). Larvae were kept under the same environmental conditions as adults and fed a porcine liver powder: brewer’s yeast mixture (2:1, wt:wt). Larval rearing water was dechlorinated using a standard aquarium dechlorinating agent.

### 2.2. In Vitro Feeding/Bioassay System

An *in vitro* bioassay system was developed (shown in Figure S2A) to blood feed mosquitoes for routine colony maintenance and to bioassay the barrier materials for bite resistance. The major components of the system are a blood-feeding reservoir, Plexiglas^®^ cage, and a circulating water bath for regulating the temperature of the blood. The blood-feeding reservoir is designed to contain the blood, fix a feeding membrane over the blood, and fix barrier materials on top of the feeding membrane for bioassays [20]. Briefly, the blood reservoir (16.5 cm length × 3.5 cm width × 0.5 cm depth) was produced with a hand-held router from a rectangular block of Plexiglas^®^ (28 cm length × 5.5 cm width × l cm thickness). A hole (4 mm diameter) was drilled at the center of the top and bottom edge through the plastic into the blood reservoir. A tap was used to cut threads into the plastic so that a valve could be screwed into the top and bottom holes. Two holes (each 4 mm diameter) were drilled from the bottom edge of the device through the plastic to the blood reservoir. A loop of stainless-steel tubing (3 mm diameter) was placed into the blood reservoir, and the tubing was inserted through the holes so that the cut ends protruded out of the plastic. Epoxy cement was used to seal the tubing in place inside the blood reservoir of the device. The ends of the tubing were connected to a circulating water bath to heat the blood.

For blood feeding, a transparent collagen film (product code 894010.95; Devro, Inc., Columbia, SC, USA) was hydrated in distilled water and stretched over the top of the device. A gasket, cut from a sheet of cork-rubber composite (Fel-Pro, part no. 3019; AutoZone, Raleigh, NC, USA) was placed on top of the collagen film. A rectangular piece of plastic (3 mm thick) the size of the blood-feeding device was then placed on top of the gasket. The central area of both the rubber gasket and plastic frame was removed so that the collagen film is fully exposed to the mosquitoes. Metal binder clips hold the gasket and frame in place on top of the blood-feeding device, preventing leakage of blood. A 30 mL syringe filled with blood was then attached to the valve that was screwed into the top hole of the blood-feeding device. With the device tilted at a slight downward angle, the blood was slowly transferred into the reservoir. The valve attached to the bottom of the device was opened to allow air displacement as the blood is added. When the device was filled with blood, both valves were closed, and the circulating water bath was started to warm the blood to 35 °C.

The barrier materials (for example, the plastic blocks shown in Figure S2C; the barrier materials tested are in toto listed in Table 1) to be evaluated for bite resistance were cut exactly to fit over the collagen film within the plastic frame. The test area for the in vitro bioassay was the same as that for the arm-in-cage studies discussed later. Masking tape, placed around the inner edges of the plastic frame, slightly overlaps the barrier. In this way, mosquitoes are prevented from gaining access to the collagen film by probing around the edges of the barrier. The blood-feeding device was inserted into a Plexiglas^®^ bioassay cage (30 cm square on each side; Figure S2A) which contains mosquitoes for feeding (with the barrier material absent) or bioassay (when the barrier material is in place). For routine colony maintenance, the feeding membrane was not covered with barrier materials.

Prior to testing the barrier materials and inserting the blood-feeding device into the cage, 100 *Ae. aegypti* females were transferred to the bioassay cage (Plexiglas^®^, 30 cm on each side). Mosquitoes were starved overnight (sugar water removed from their rearing cage; females not blood fed) prior to testing. Female mosquitoes were 6–7 days of age (post emergence). Porcine blood obtained from a local abattoir was used in our bioassays. At the time of blood collection, sodium citrate was added as an anticoagulant. Just prior to initiating the bioassay, ATP (Sigma) was added to the blood (2.5 mg/mL) as a phagostimulant [20]. Each bioassay was conducted for 10 min., during which the number of times females landed and probed the barrier material was counted. A single event was recorded if a female landed and then inserted or attempted to insert her proboscis into the barrier material, regardless of whether the female probed multiple times after landing. A video recording was made of each bioassay so that the mosquitoes’ responses to the surface of each barrier and probing behavior could be studied. At the end of the exposure period, mosquitoes were removed and killed in a freezer. Subsequently, each mosquito was crushed on a sheet of white paper to determine if she was able to probe through the barrier and obtain a blood meal. Blood spots on the paper were counted, and the percentage of mosquitoes that were blood fed was calculated based on the total number of mosquitoes released into the cage. The *in vitro* bioassays were repeated for each barrier material a minimum of 3 times. For routine blood feeding for colony maintenance, the number of mosquitoes in the cage was variable (50 to 200), and the feeding time extended until all of the mosquitoes that want to feed have time to feed to repletion. All bioassays and mosquito adult feeding, including the *in vitro* and *in vivo* (described later) tests, were conducted in the mosquito insectary laboratory at the Dearstyne Entomology Building of NC State University, at a temperature of 27–29 °C and 75–80% humidity. All tests were conducted during the photophase under florescent lighting.

### 2.3. In Vivo Bioassay for Bite Resistance

Measurement of the *in vitro* mosquito-bite resistance of the barrier materials was standardized in terms of the apparatus architecture (dimensions and exposed area of the feeding membrane) and blood-feeding conditions. Similarly, for the *in vivo* studies, the dimensions of the bioassay cage and cloth area exposed for mosquito probing were the same. Our IRB for the *in vivo*, arm-in-cage studies required us to demonstrate *in vitro* bite resistance of greater than 80% for the barrier materials before conducting an *in vivo* test on the same barrier material. This restriction was to limit the potential number of mosquito bites received by the human subject. *In vivo* tests using human subjects is a more rigorous test of a fabric’s bite resistance because of the volatile attractants emitted from the skin. *In vivo* testing is critical to understanding whether a textile will prevent mosquito bites. Therefore, validation of our predictive model and development of textiles for garment construction (discussed later) required *in vivo*, arm-in-cage studies.

Arm-in-cage studies (apparatus used shown in Figure S3A) were conducted with informed consent using a protocol for use of human subjects in research approved by the NC State University Institutional Review Board (IRB #2925) [21]. The assay methodology was designed to mimic a textile worn on the forearm with the fabric in close contact with the skin. Odorants and heat from the skin can diffuse through the fabric attracting mosquitoes seeking a blood meal.

The sleeve device (Figure S3A), constructed from bioassay textiles, exposed the cloth surface through an opening that was identical in size as was used in the *in vitro* assays. The sleeve was constructed from a polyvinyl-coated roofing membrane, Samafil^®^ (Sika Corp., Canton, MA, USA). The sleeve was cut into a trapezoidal shape to fit a human arm and with a 16.5 cm × 3.5 cm opening in the center that corresponds to the size and shape of the opening in the *in vitro* blood-feeding device described earlier. A plastic frame was riveted to the sleeve to keep the exposure area of the textile from deforming when the sleeve was attached to the forearm of the study participant.

In total, 100 unfed, nectar-starved *Ae. aegypti* adult females were transferred to a bioassay cage 10–30 min before being assayed, as described earlier for the *in vitro* assay. The textile to be assayed was laid over the underside of the forearm of the study participant. The sleeve was laid on top of the cloth and attached to the participant’s forearm with Velcro^®^ straps. The hand of the participant was then covered with a nitrile glove to prevent mosquito bites on the hand. The bioassay was started when the participant inserted his/her arm through a cloth sleeve into the bioassay cage. An observer counted the numbers of mosquitoes landing on the cloth and probing during a 10 min exposure period, and in some cases video recordings were made of the inserted arm only as needed for further documentation. After the bioassay was terminated, mosquitoes were examined for blood feeding by crushing them on white paper as previously described for the *in vitro* assay. Blood spots on the paper were counted, and the percentage of mosquitoes that were blood fed was calculated based on the total number of mosquitoes released into the cage. The mosquitoes used, mosquito conditioning, the number of mosquitoes, and level of replication were the same as that described for the *in vitro* assay.

### 2.4. Walk-in-Cage Studies of Whole Garments

A garment is composed of integrated fabrics and seams that have various rectilinear and curvilinear pattern pieces needed to conform to differing human body shapes. The gap distance between the garment and the skin varies throughout the body and can change with posture along with textile stretching, all of which can affect bite resistance. These factors affect the fabric performance regarding mechanical bite resistance and comfort, which can only be evaluated through whole-garment testing. Walk-in-cage studies provide a method for testing garments under quasi-field conditions with higher mosquito-bite pressures. We also avoided disease risks to human subjects that might occur using wild mosquito populations in a field test.

Garments (Figure S6A,B, described later in detail, and all the garments tested are listed in Table 1) were tested in a walk-in enclosure (2 m height × 4 m length × 4 m width) constructed from polypropylene screens (mesh size 1.8 mm; Lumite Company, Alto, GA, USA) that were sewn together to form a cage. The test cage had a zippered opening and was supported with a 2 inch × 4 inch wooden frame. The bottom edges of the panels were taped to the cement floor to prevent mosquitoes from escaping. The cage was covered with white bed sheets and then an outer layer of black plastic to block external light. Light inside the cage was provided by a single 35 W fluorescent tube placed at each corner suspended from the ceiling. Prototype garments were worn by a human subject with informed consent with an approved research protocol (IRB# 9075) from the NC State University Institutional Review Board. For the prototype base layer garment, the subject’s head and neck were protected by a bee veil, the hands were covered by nitrile gloves and the feet covered with shoes. Each pant’s leg was taped to the shoe to prevent biting at the margin between the pants and shoe. For the prototype NCSU shirt, the subject wore three pairs of pants that combined were 100% bite proof; otherwise everything was the same as for the base layer.

At the beginning of the trial in the bioassay cage, 200, 6–7-day-old, unfed adult female *Ae. aegypti* were released by the test subject. The condition of the mosquitoes was described earlier. In the bioassay cage, the subject stood motionless with arms at her/his sides for 10 min and then sat with arms crossed for an additional 10 min on a waist-high stool (no back support). In a sitting position, the fabric was stretched at the knees, elbows, and shoulders. These two postures mimicked how a garment would be worn for mosquito protection. The postures caused the garment to deform, changing the gap distance between the fabric and skin on different parts of the body, thus potentially affecting bite-resistance performance. Assays were conducted during the photophase at 25–28 °C and a relative humidity of approximately 30–40%. At the end of each trial, the subject exited the bioassay cage, and all mosquitoes were collected with a mechanical aspirator and killed in a freezer. After removing the garment, the test subject’s skin was examined for mosquito bites with the assistance of another researcher. Areas of the body where bites occurred were recorded so that the corresponding areas of the garment could be reinforced to prevent bites in subsequent prototypes. Mosquitoes were collected, frozen, and examined for blood feeding by crushing them on white paper, as described earlier. Each garment was evaluated in a minimum of three separate trials conducted on different days.

### 2.5. Model Rationale and Mosquito Morphometrics

Blood feeding of mosquitoes on humans involves physical interactions between the mosquito’s external morphology associated with the head and exposed skin, requiring a combination of insect behaviors allowing the mouthparts to penetrate the cornified, squamous epithelium and insert into the host blood vessels near the skin surface. When a textile is placed over the skin, the fabric restricts access to the skin and affects mosquito landing and probing behaviors. This creates another compliment of physical interactions between the textile and the mosquito that affects differently how the mosquito also interacts with the skin below. These physical parameters of the mosquito’s head and mouth parts impose three-dimensional limits, defined by their shape and size, on a mosquito’s ability to penetrate the textile and the skin. Understanding these limits and the mechanics of biting affected by the physical structure of cloth and the morphometrics of the mosquito’s feeding structures can be used to develop textiles to optimally resist blood feeding, as well as providing optimal comfort without the need for insecticides or repellents.

The mosquito proboscis (Figure S1A,B) is a collection of interlocking needle-like mouthparts (stylet in shape) covered by a sheath, the labium. The stylets consist of the labrum (Figure S1C,D), a pair of mandibles, a pair of maxillae, and a hypopharynx extending from the floor of the mouth between the mandibles and maxillae. The rigid, pointed labrum tip is shown in Figure S1D and is the first part of the proboscis that makes contact with skin to initiate biting. The other mouth parts are used to advance the insertion into the skin and for channeling blood to the mouth. Preventing labrum penetration and/or contact with the skin prevents blood feeding.

Our model to describe the physical interactions between a mosquito and a barrier material is divided into three Cases that represent the process of fabric penetration to obtain a blood meal and how the mosquito interacts with different textile surfaces. For our Case 1 model (Figure 1E), the dimension of the labrum (the largest mouthpart needed for penetration of the skin and blood feeding) is a critical attribute of the mosquito’s mouthparts. To measure its dimensions, the labrum from 20 adult female mosquitoes (described before) was dissected using needle-point forceps, then gold coated using a SC7620 Mini Sputter Coater (Quantum Design GmbH, Darmstadt, Germany), visualized using a Phenom G1 desktop scanning electron microscope (SEM; Thermo Fisher Scientific Inc., Waltham, MA, USA) in the Phenom SEM and Forensic Textile Microscopy Laboratory at NC State University, and the measurements of maximum labrum diameter (D), labrum tip angle (α), and tip length (*L*_tip_) taken from these images. To avoid body shrinkage from dehydration, the mosquitoes were killed by freezing, and the mouth parts were quickly dissected and gold coated.

For the model for Case 2 and Case 3 (Figure 1E), 20 adult females were used for measurements of the head diameter (*D*_head_) and antenna length (*L*_antenna_), not including the flagella branches and proboscis length (*L*_proboscis_), using a Nikon SMZ-1000 Zoom Stereo Microscope fitted with an ocular micrometer (Nikon Metrology, Inc., Brighton, MI, USA) in the Phenom SEM and Forensic Textile Microscopy Laboratory at NC State University. To avoid body shrinkage from dehydration, the mosquitoes were killed by freezing and then morphometric measurements were immediately taken. The mosquito anatomy that was measured is shown in (Figure S1B,C).

### 2.6. Model Development

Based on observations of mosquito probing and biting behavior, we hypothesized that the morphometrics critical for blood feeding were associated with the head size and length, the relationship of the antennae to the head, and the length and diameter of the labrum. Based on these assumptions, there were three rationales on how a textile might be used to prevent penetration of the skin: (i) a barrier that is thick enough to prevent the labrum from reaching and penetrating the skin; (ii) a barrier with small enough pores that prevented the labrum and/or the head from penetrating the surface of the textile; and (iii) combinations of (i) and (ii). The boundaries for thickness based on our morphometrics were set from 0 to 2.95 mm (the sum of the head diameter and proboscis length) and the boundaries for pore diameter were from 0 µm to 1.8 mm (the sum of the antenna length and head diameter). Due to the complex geometry between the head and proboscis, we specified three cases to achieve a bite-resistant structure: pore diameter smaller than the diameter of the labrum, pore diameter smaller than the head diameter, and pore diameter smaller than the sum of the head diameter and antenna length. In those cases, each pore diameter has a specific thickness determined by the geometry of the mosquito mouthparts, head, and antenna that would impact biting.

**Figure 2:**
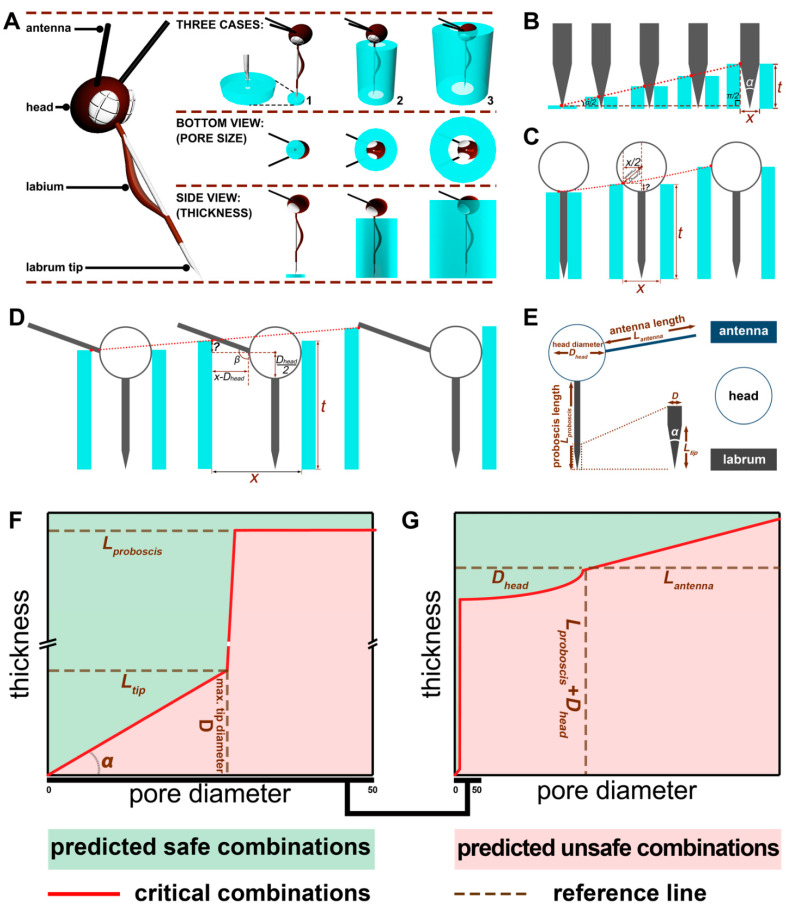
Bite-resistance model development. (**A**–**D**) Ice green vertical bars are the textile barrier, and the red dotted line the critical combination of pore diameter and thickness of the textile barrier. (**A**) Three cases that prevent mosquito biting based on mosquito anatomy. (**B**) Case 1—the pore diameter is smaller than the labrum diameter. (**C**) Case 2—the pore diameter is larger than the labrum diameter but smaller than the head diameter. (**D**) Case 3—the pore diameter is larger than head diameter but smaller than the sum of the head diameter and antenna length. (**E**) Abbreviations for length and diameter of the mosquito anatomy. (**F**) Zoomed-in view of the Case 1 model. (**G**) Case 1, Case 2, and Case 3 model predictions. Brown dotted lines in (**F**,**G**) are the critical parameters measured from the anatomy of the *Ae. aegypti* female in Figure S1 that define the three cases’ combinations of porosities and thicknesses of the textile.

The bite-resistance model describing the relationship between the pore diameter and thickness of a textile barrier is shown in Figure 2B–D. In Case 1, the critical trajectory of the combination of pore diameter and thickness is the hypotenuse of a right-angled triangle (the longest side) of the labrum. In Case 2, the critical factor is the arc determined by the head shape. In Case 3, the critical factor is a straight line governed by the antenna. Based on this geometry, we defined the mathematical relationships for each case.

### 2.7. Materials for Model Validation

#### 2.7.1. Stable Structures

Due to the sophisticated interlacement and entanglement of the fibers [22], most textiles have irregularly distributed pores of different shapes and area and an uneven thickness. In terms of the latter, a textile never has an absolute planer surface. Because of this variability, relating textile structure to bite resistance is not precise. This is further complicated by the large variety of possible textile structural parameters that can be selected, including yarn denier, covering rate, surface roughness, weave or knitting density, etc. Therefore, the use of a textile with a single pore shape and size and a single, fixed thickness is challenging and requires testing a vast number of iterations using different textile production methods. Instead, our first step in model validation was the use of stable structures.

For Case 2 and Case 3 conditions, we simulated a porous fabric with rigid polypropylene plates (Figure S2C) with bored holes of varying diameters that were distributed in uniform patterns on each plate where we could simulate precise pore shapes (circular), pore areas, and textile thicknesses. The size of each polypropylene plate was fixed at 14.5 cm × 3.4 cm to fit into the *in vitro* bioassay device described earlier. Based on mosquito morphometrics, we focused on 3 different pore diameters which (i) included the head (1.25 mm); (ii) partially excluded the head (0.8 mm); and (iii) completely excluded the head (0.5 mm). Those plates were produced by a combination of 3D printing to obtain the correct thickness and computer numerical controlled (CNC) machining to obtain a specific pore size and number of holes. First, a plain mold was printed on a 3D printer (Objet Connex350, Edward P. Fitts Department of Industrial and Systems Engineering, NC State University, Raleigh, NC, USA) to the desired thickness. Then the pre-designed pattern was processed on a CNC machine to obtain holes with precise diameters that would mimic a porous textile. A series of prototype spacers (S = plastic spacer; S1, S2…, S8, listed in Table 1) were made at different combinations of pore sizes and thickness, which spans Case 2 and Case 3’s safe and unsafe combinations. As shown in Figure S5C,D, S1 is 2.1 mm thick, with a 0.5 mm pore diameter; S2 2.1 mm thick, with a 0.8 mm diameter; S3 2.5 mm thick, with a 0.5 mm diameter; S4 2.5 mm thick, with a 0.8 mm diameter; S5 2.5 mm thick, with a 1.25 mm diameter; S6 2.72 mm thick, with a 0.8 mm diameter; S7 2.75 mm thick, with a 1.25 mm pore diameter; and S8 3 mm thick, with a 1.25 mm diameter.

The holes in each plate were of uniform diameter. The ratio of open space (from the pores) to closed space (from the solid surface) was held constant in these studies. If the number of pores per plate was held constant but pore diameter increased, there would be an increasing probability that the probing mosquitoes would encounter a pore by chance alone. Furthermore, differences in the open area across a plate affects the amount of mosquito attractants (heat and odor [23]) penetrating through the holes in the plate. These attractants can affect landing and biting rates. Accordingly, as pore diameter was increased, a smaller number of pores were needed per plate. If the number of pores is designated as *N* and the diameter of a pore is designated as d with a unit of cm, the percentage of open area in a spacer should be a constant *C*, as shown in Equation (1):

(1) $$C=\frac{N\cdot \pi {\left(d/2\right)}^{2}}{14.5\times 3.4}$$

To keep the probability of a mosquito encountering a pore constant, the equation shows that the number of pores *N* in a spacer is inversely proportional to the square of the diameter of a pore, d. From the equation, the value of *N* was 572, 1396, and 3574 for pore diameters at 1.25, 0.8, and 0.5 mm, respectively.

For the Case 1 barriers, constructing thin plastic plates of ~75 µm or less by 3D printing was not possible. The thickness was too variable across the area of the plate. Furthermore, drilling small pores of ~28 µm or less by drilling across a thin plastic plate was not possible. To achieve the operational parameters needed to test the Case 1 model, commercially available Saatifil^®^ polyester woven filtration fabrics were used (W = woven; W1, W2, W3, and W4, listed in Table 1) (shown in Figure S2B). In Figure S5A,B, W1 is 52 µm thick with a 25 µm pore dimeter, W2 is 60 µm thick with an 18 µm diameter, W3 is 58 µm thick with 14 µm pores, and W4 is 86 µm thick with 8 µm pores. These fabrics had square pores produced when the polypropylene monofilaments were woven in a plain weave pattern. The size of each woven fabric was 14.5 cm × 3.4 cm to fit into the *in vitro* bioassay device already described. We evaluated the bite resistance of four monofilament woven fabrics and the plastic blocks using the *in vitro* bioassay described earlier.

#### 2.7.2. Knitted Textile Structures

To further validate our model for flexible textiles (T = textile materials; Table 1), we constructed fabrics including one predicted unsafe and one predicted safe according the model for each Case.

Case 1: The Case 1 fabric (T1; Figure S2D) was an ultra-fine synthetic knit of 80 percent polyamide of 20 denier count (a unit of measure for the linear mass density of fibers, the mass in grams per 9000 m of the fiber) and 20 percent elastane of 15 denier count and has a weight of 82 g/m^2^. Its pattern was a jersey plated knit structure of 78 wales and 104 courses per inch and with a pore size between 32 and 42 µm. The pore diameter of T1 in Figure S5F was larger than the diameter of the mosquito labrum. To reduce the pore diameter based on our Case 1 model, we used a 1 m-wide, laboratory oil-heated Stork laminator (Stork GmbH, Bavaria, Germany) to heat set the fabric in the Dyeing and Finishing Pilot Plant at NC State University. The temperature was 190 °C (lower than *T*_g_ of the polyamide) with a 120 s duration. It was found that the pore diameters of the fabric (T2) was reduced by this treatment to 10 µm from 16 µm and the thickness reduced to 0.26 mm, as shown in Figure S5E,F (predicted to be safe by the Case 1 model).

Case 2: 3D spacer fabrics (T3, T4: satin weave + pillar stitch; Figure S2E) were produced on a double-needle bed, Raschel warp knitting machine with six guide bars (Rius Mini-tronic Raschel Warp Knitting Machine, RIUS-COMATEX, Barcelona, Spain) in the Knitting Laboratory at the Wilson College of Textiles at NC State University. The material consisted of 100% polyester (Huizhou City Meilin Textile Co., Ltd., Huizhou, China). For the pile yarn, a 33 dtex (a unit of direct measure of yarn linear density, grams per 10 km of yarn) monofilament was used. The outside surface was made with 55 dtex multi-filaments. Both multi-filaments contained 36 filaments, respectively. To make variations in the design, the take-up speed was changed. Hence, the stitches per cm and the thickness would change. The T3 fabric was made by a 700% take-up speed, and the T4 one made by a 900% take-up speed. The combination of thickness and pore diameter of the T3 (Figure S5E,F) was predicted unsafe while that of T4 was predicted safe.

Case 3: The 3D spacer (warp) knit fabric for Case 3 had the same pattern and materials as the Case 2 fabrics, which were produced on the same Raschel warp knitting machine. Case 3 fabrics T5 and T6 (Figure S2E) were produced at 1500% and 1200% take-up speeds. The T5 thickness was 2 mm with a pore diameter of 940 µm. T6 was 3 mm and 770 µm, respectively (Figure S5E,F). Based on the model prediction, T6 is a safe material that should resist mosquito bites.

We evaluated the bite resistance of the Case 1, Case 2, and Case 3 fabrics using the *in vitro* bioassay system described earlier. All the materials used in the model validation, as listed in Table 1, including the woven textiles, plastic plates, and knits, were white in color to avoid potential mosquito preferences in landing and biting based on color differences.

### 2.8. Finite Element Model for Proboscis Penetration

**Figure 3:**
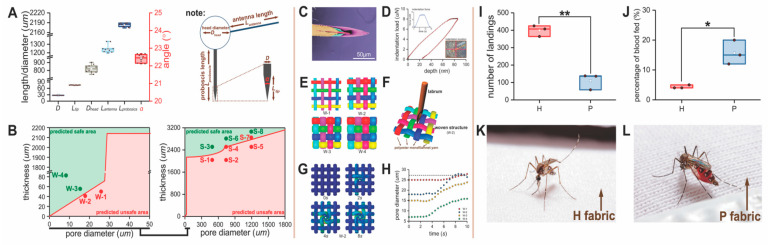
Mosquito morphometrics, model prediction based on mosquito morphometrics, impact of fabric distortion on biting, and comparison of non-insecticide versus insecticide-treated textiles for bite resistance. (**A**) Measured parameters of mosquito anatomy (average value calculated from 20 measurements for each parameter). (**B**) Model prediction of safe and unsafe woven filtration fabrics (left graph) and plastic plates (right graph). See Figure 4 and Table 1 for the in vitro bioassay results and Table 1 for the barrier abbreviations and whether the prediction was correct. (**C**–**H**) A demonstration of the textile structure failing to resist the mosquito bite at the critical boundary between safe and unsafe (Figure 3B) due to enlargement of the pore under labrum penetration. (**C**) Tip of proboscis. To measure the resistance to proboscis penetration, the mechanical property of the labrum (pink color) was measured. (**D**) Nanoindentation curve of the labrum. The elastic modulus was 1.35 GPa calculated by the load–depth curve. (**E**) Illustration of four weave patterns (W = Case 1 validating Woven structures, W1 to W4). (**F**) Model of the W2 fabric under pressure from proboscis penetration. The elastic modulus and Poisson’s ratio of the polyester monofilament used in this model was 2.16 GPa and 0.3, respectively, evaluated on the MTS^®^ tensile tester with 2 cm gauge and 5 mm/min speed. (**G**) Deformation process of W-2 subjected to proboscis penetration. (**H**) Variation of the pore diameter caused by proboscis penetration. The black dashed line is the maximum proboscis diameter. Pore diameters of W1 and W2 increased beyond this critical value, and thereby failed to resist proboscis penetration. (**I**,**J**) Arm-in-cage bioassay results for fabrics H and P: (**I**) difference in the number of landings was statistically significant at *p* < 0.01; (**J**) difference in the percentages of blood-fed mosquitoes was significant at *p* < 0.05. (**K**) Mosquitos failing to probe through the H fabric because of its small pore size (obvious proboscis bending trying to push through the H fabric). (**L**) Blood-fed female after successfully penetrating through the P fabric.

In addition to our Case 1–3 conditions, we needed to investigate the point of contact of the proboscis to a textile surface and how this specific interaction might impact our prediction of penetration (especially relative to the Case 1 model). The finite analysis model was necessary because for Case 1, predictions based on labrum diameter alone were not 100% correct in predicting blood feeding when approaching the boundary between safe and unsafe textiles (Figure 3B). This result suggested additional physical interactions might be in play that were important in preventing biting. Finite Element Analysis was conducted for a woven versus a knitted structure to examine two possible scenarios for micro-deformation. The woven model was used for investigating the interaction of the woven structures and the knit to understand the role of stretching.

Structural parameters of woven and knit structures were obtained by the calculation of fabric thickness, weave density and spatial axial distribution [11,24], which were then imported into SolidWorks^®^, a computer-aided design program, for establishment of a geometrical model. The boundary conditions of both the woven and knit model were set to periodical boundary conditions [25] for approximating a large (infinite) fabric piece by using a small fraction of the piece. Since only a small force is applied in both scenarios, the mechanical property for the knit and woven model can be treated as linear elastic materials.

To simulate the pore deformation of the woven structure, a virtual labrum with the same mechanical properties and shape of a real mosquito labrum was used to penetrate the woven fabric. This virtual labrum will be discussed more later. The test was analyzed using the software suite SIMULIA Abaqus/Explicit 6.14. The elastic modulus and Poisson’s ratio of the polyester monofilament used in this model were 2.16 GPa and 0.3, respectively.

For modelling the virtual labrum, we needed the fundamental mechanical properties of the proboscis. Because of its small size, traditional methods to measure tensile and compression [26] were not possible. Alternatively, the elastic properties of the proboscis were determined with a Bruker Hysitron TI980 Triboindenter (in the NC State University Analytical Instrumentation Facility). The measured location and load–depth curves are shown in Figure S7C. The elastic modulus of the proboscis can be achieved by the initial part of the recovery curve [27].

To simulate the pore deformation of the knit structure, virtual tensile forces were applied to the model in the course and wale directions (Figure S7B), and the simulated deformations compared with the real fabric deformation (Figure S7D,E). The elastic modulus and Poisson’s ratio of the blended yarn used in this model were 1.08 GPa and 0.21, respectively. The knit model was validated using the experimental tensile data (Figure S7C) to ensure they have an equivalent mechanical property as the real knit fabrics.

### 2.9. Prototype Bite-Resistant Fabrics Tested for Garment Construction

Three knitted fabrics (H, B, S; Table 1 and Figure S3B–D) were developed as component textiles for garment construction. They were selected from a dataset of candidate bite resistant fabrics that were predicted safe by our bite-resistance model. These textiles were assayed using arm-in-cage bioassays since the goal later was to test them in garments on human subjects in walk-in-cage studies.

Case 1 H. The Case 1 fabric H (the high-density fabric, H; Figure S3B) was an ultra-fine synthetic knit of 80 percent polyamide of 20 denier count and 20 percent elastane of 20 denier count and had a weight of 96 g/m^2^. Its pattern is a jersey plated knit structure of 84 wales and 112 courses per inch and with a pore size between 20 µm and 28 µm, allowing air passage but preventing mosquito biting. It had a high elasticity of 400% stretch in the course direction and 160% stretch in the wale direction (Figure S7C). The H fabric has a more elastane content and smaller pore size compared with T1, which came from the same knitting technology. It was made into a base layer in the following section “construction of protective garments”. Although the H fabric was not a 100% bite-resistant material due to an irregular pore distribution in the knit pattern, when combined as a base layer with military issued garments, a 100% bite resistance was possible in whole-garment testing.

Case 1 B. Fabric B (a bonded fabric; Figure S3C) is the combination of two layers of H fabric that was made by applying a small dot pattern of dry low-melt adhesive (CG-1698 polyurethane adhesive, Chemix Guru Ltd., Taichung, Taiwan) to one surface and then feeding the two fabrics back-to-back together applying pressure using heated drums (temperature 120 °C, duration 20 s). The two fabrics are fused together at regular intervals, and then the adhesive dots subjected to cool circulating air for 24 h to eliminate volatiles that might affect mosquito biting. The paste dot application procedure is particularly gentle to the substrate, and the wide range of options for formulating the paste provides the user flexibility in the application procedure. The relative nature, drape, porosity, and flexibility of the fabric is maintained, and this method only adds approximately 5% to the total weight. The B fabric is highly stretchable and demonstrated high mosquito bite resistance, which makes it suitable to being used as an outer protective garment.

Case 2 S. The S fabric (3D spacer fabric; Figure S3D) was a commercially available 3D warp knit spacer fabric (Production ID: 34836, Springs Creative Products Group, LLC, Rock Hill, SC, USA) that was predicted safe for bite protection using our Case 2 model. The surface (top and bottom) yarns are PA filament tows, and the pile yarns used in the middle layer were PA monofilaments. The surface patterns are shown in Figure S3D. The S fabric had a stable structure with large openings outside that allowed air flow into and under the garment, thereby transporting of heat and sweat out.

Case 3. Case 3 fabrics were translucent due to their large pores and not practical when used alone for typical garments where human body parts need to be covered and not seen by others. Therefore, we did not use the Case 3 fabrics to assemble a garment. This is not to say this fabric does not have uses for mosquito protection in parts of the body where it is ok to show the skin or as a cover at the beach or in the tropics where there are mosquitoes and also high thermal challenges to the body. The materials could also have uses for garment ventilation in specific areas of a garment.

Base on the color requirement for military garments, the H fabric was dyed to a light brown color before assembly into the base layer. B and S were dyed to a camo color before assembly into the military-style shirt (NCSU shirt).

### 2.10. Textile Structural Analysis

As mentioned before, fabric pore size and thickness are two critical factors in our model that determined bite resistance. Hence, it was important to measure these variables accurately. Pore areas in textile materials, especially in knitted fabrics, have irregular shapes due to complex fiber configurations. Pores with an elliptical shape often failed to resist mosquito bites even though the pore openings were narrower than the proboscis in one direction. We also found irregular pore openings were difficult to measure accurately and were not informative to our model. Therefore, we assumed pores to be circular, and we measured pore diameter across the widest area of fabric pores so that the model would reflect a worst-case scenario.

Pore diameter was measured (Figure S4) with a digital microscope (Bausch & Lomb, Monozoom-7 Zoom Microscope), and images analyzed using ImageJ software, an open-source image-processing program designed for analyzing multidimensional images [28]. Based on Feret’s diameter, the width of the pore along its longest direction, a frequency distribution of the pore diameters, and a fitting curve were obtained. From the peak of the fitting frequency distribution, we picked three maximum diameters for each fabric to calculate the average maximum pore diameter (4 images were captured for each fabric, a total of 12 measured values). Fabric thickness, measured with a Thwing-Albert ProGage Thickness Tester (Thwing-Albert ProGage instrument company, West Berlin, NJ, USA) was averaged over 10 tests, using standard methods for assessing textile thickness, as described in the ASTM D1777 guidelines [29]. The procedure of measuring pore diameter is shown in Figure S4, and the values of the measured pore diameters and fabric thicknesses are shown in Figure S5.

### 2.11. Comparison of the Non-Insecticide and Insecticide-Treated Textiles

Before garment construction, it was prudent to understand how our bite-resistant, non-insecticidal textiles performed relative to a leading brand of insecticide-treated cloth. We compared the bite resistance of the H fabric with a commercially available permethrin-treated T-shirt fabric (P = permethrin, listed in Table 1), which was cut from an InsectShield^®^ T-shirt (RN149846, Insect Shield, LLC, Greensboro, NC, USA) purchased from a local retail store. The fabric was 70% cotton and 30% polyester and cut into 14.5 cm × 3.4 cm for the arm-in-cage (in vivo) bioassays.

### 2.12. Construction of Protective Garments

Based on the predictions of our model, three types of fabrics were used as bite resistant materials: a superfine knit fabric (H), a double-layer bonded knit fabric (B), and a knitted 3D spacer fabric (S), as shown in Figure S3B–D. Two types of garments were produced: a base layer and a military-style combat shirt, as shown in Figure S6A,B.

Base layer (Figure S6A). A form-fitting undergarment was constructed consisting of an upper body, form-fitting garment having a torso section and arm sections made from the Case 1 fabric H. The garment was fitted with an elastic neck cuff secured to define a neck opening for the torso section; an elastic waist cuff secured to define a waistband around the torso section; and a pair of elastic wrist cuffs disposed at an outer terminus of each of the arm sections. The ensemble also included a lower-body, form-fitting garment having a waist section and left- and right-leg sections made from the same textiles as previously described for the shirt. The pants were fitted with an elastic waist cuff secured to define the waistband around the waist section and a pair of elastic ankle cuffs disposed at the terminus of each of the left and right leg sections. The cut and sewing of this garment were conducted in the Fashion Studio at the Wilson College of Textiles at NC State University. The garment was unwashed and tested in walk-in-cage studies (described earlier).

NCSU shirt (Figure S6B). A long sleeve shirt was constructed as an upper-body, form-fitting garment. The shirt consisted of Case 1 B and Case 2 S fabrics. The incorporation of the B fabric provides extensionality and bite resistance, while the use of the S fabric brings breathability, pressure release, and bite resistance to the shirt. The S fabric was designed into the sections of the shoulders, chest, back, and elbow of the garment, and the remainder of the shirt was the B fabric. The cut and sewing for this garment were conducted in the Fashion Studio at the Wilson College of Textiles at NC State University. The garment was unwashed and tested in walk-in-cage studies (described earlier).

Both garments were sewed on an MF 7924 cover stitch sewing machine (JUKI, Singapore) and locked on a DDL-8700-7 lockstitch machine (JUKI, Singapore). The sewing thread was 100% polyester (RCL, model: RCLJ-ST-W, Wuxi, China). The seams were bite resistant in the walk-in-cage bioassay, since there was a two-layer overlap of the textile at the connections between the two pieces of cloth.

### 2.13. Sweat Manikin Test for Comfort Evaluation of Garments

In the Textile Protection and Comfort Center of NC State University, a sweating manikin was used to evaluate the thermal insulation and breathability of the garments [30] (Figure S6D). The test instrument is composed of a manikin, an environmental chamber, an ambient detector, a power supplier, a water reservoir, and a pump.

Comparisons were made with a commercially available base layer garment (Under Armour^®^ men’s base 1.0 crew, model: 1281079, Under Armour Inc., Baltimore, MD, USA) and a military-issued combat shirt (Winter Army Combat Shirt Test, made in the USA by NIB/NCW, Figure 5A). The comparison garments had similar material characteristics and knit patterns to our garments. Each comfort evaluation was replicated three times, after which average values were calculated.

Manikin zones (a group of thermal-sweat elements on the manikin) were measured for thermal resistance and evaporative resistance. The standard method for measuring thermal resistance is described in ASTM F1291 and was followed. Test conditions for thermal resistance were 20 °C, 50% relative humidity, and a 0.4 m/s air speed with a 35 °C skin temperature. The measurement standard of evaporative resistance was ASTM F2370. Test conditions for evaporative resistance were 35 °C, 40% relative humidity, and a 0.4 m/s air speed with a 35 °C skin temperature. The following parameters were obtained from the manikin test: *R*_t_ (°C·m^2^/W), the total thermal resistance provided by the manikin, garment ensembles, and air layer; *R*_et_ (kPa·m^2^/W), the total evaporative resistance provided by the manikin, garment ensembles, and air layer; *R*_cl_ (°C·m^2^/W), the instinct thermal resistance provided by the garment ensembles only; *R*_ecl_ (kPa·m^2^/W), the instinct evaporative resistance provided by the garment ensembles only; *I*_t_ (clo), the total insulation provided by the manikin, garment ensembles, and air layer (higher *I*_t_ values mean the garment has a higher thermal insulation property that would not be desirable in warm weather for a bite-resistant fabric); *i*_m_, the moisture-heat permeability through the fabric on a scale of 0 (total impermeable) to 1 (total permeable) normalized by the permeability of still air on the naked skin; and *Q*_predicted_ (W/m^2^), the predicted heat loss potential, which gives a predicted level of the total amount of heat that could be transferred from the manikin to the ambient environment for a specified condition. The *Q*_predicted_ incorporates thermal and evaporative resistance values to calculate the predicted levels of evaporative and dry heat transfer components for a specific environmental condition. In this case, the specified environment was 25 °C and a 65% relative humidity. The overall *Q*_predicted_ under these conditions was calculated by adding the predicted dry component of heat loss to the predicted evaporative component of heat loss and reflected the predicted total amount of heat loss possible. The test results of all parameters are shown in Table S1.

### 2.14. Data Analysis

**Figure 4:**
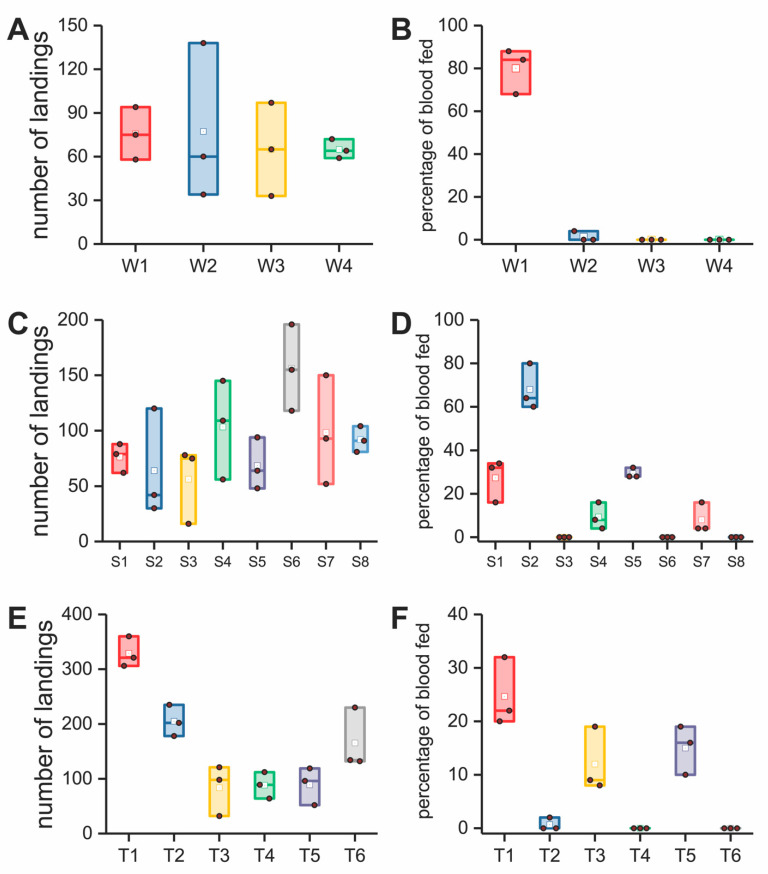
In vitro bioassay results for the woven structures, plastic plates, and knitted and knitted spacer fabrics (see Table 1 for the pore size and thickness, model prediction, and whether the prediction was accurate; Figure 3B for the position of the woven structures and plastic plates relative to the safe and unsafe barriers predicted by the model). (**A**) Number of landings on the woven structures. (**B**) Percentage of blood fed on by mosquitoes on woven structures. (**C**) Number of landings on plastic plates. (**D**) Percentage of blood fed on by mosquitoes on plastic plates. (**E**) Number of landings on knitted fabrics. (**F**) Percentage of blood fed on by mosquitoes on knitted fabrics. Abbreviations used: W1 to W4 = Case 1 woven filtration structures; S1 to S8 = Cases 2 and 3 plastic spacer blocks; T1 to T6 = Cases 1–3 knitted and spacer (3D) knitted textiles (see Table 1 for more detailed definitions of the abbreviations).

**Figure 5:**
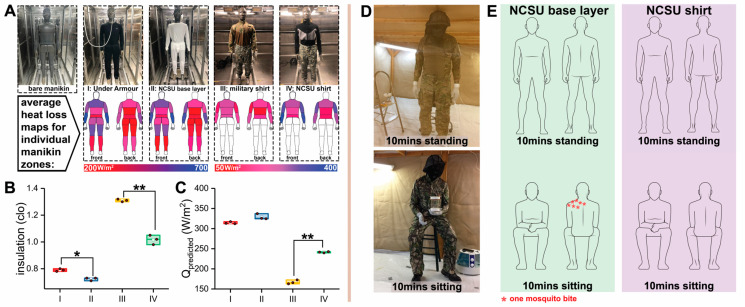
Prototype garment’s comfort and bite-resistance compared to commercially available similar garments. (**A**) Manikins equipped with various garments (I, Under Armour^®^ base layer; II, NC State base layer; III, winter army combat shirt; and IV, NC State shirt), also showing the average heat-loss maps. (**B**) Garment insulation. Since mosquitoes mostly appear in warm weather, a garment with low insulation properties is preferred. The NCSU base layer and NCSU shirt provided lower levels of insulation compared with the comparative garments tested (*p* < 0.05 and *p* < 0.01, respectively). (**C**) The *D*_predicted_ values (predicted heat loss; Table S1) for the garments tested. II the NCSU base layer showed an equivalent thermal and moisture management compared with I. IV, the NCSU shirt exhibited better thermal and moisture management compared to III (*p* < 0.01). (**D**) Walk-in-cage bioassay with 10 min standing and 10 min sitting. The container in the hands of the subject (bottom picture) housed the mosquitoes. The mosquitoes were typically released, and the test started with the person standing (note the empty container on the stool, top picture). (**E**) Walk-in-cage bioassay results for the worst-case replicate shown (* = one mosquito bite). Bites on the shoulder were observed where the most stretching of the garment occurred and bite resistance was reduced. A specially designed double layer was used in this part of the NCSU base layer which eliminated all bites in the walk-in-cage bioassay (data not shown).

All the replicated data for the assays and comfort analyses (Figure 3, Figure 4 and Figure 5 and Figure S5) were plotted in ORIGINPRO^®^ 2018 using a box plot format, a graphical format that summarizes the key statistical values. The solid brown dot in the box plot was the raw data. The height of the box represents the 25th and 75th percentiles. The whispers represent the 5th and 95th percentiles. Additional values included the median (line inside of the box) and mean (white dot) presented in the box plot. We used the mean value of each data set for our analyses.

We used one-sample Student’s *t*-tests to investigate the significance between two data sets in Figure 3I,J and Figure 5B,C The mean value of the first data set was used as the theoretical expectation. The second data set was set as the true mean. Differences in mean values were found to be statistically significant when the *p* values were greater than 0.05 (*) or 0.01 (**).

All tested materials and garments are listed in Table 1, including information on the material type, name, abbreviation, thickness, pore diameter, model prediction, and bioassay validation. Values of thicknesses and pore diameters are the mean values calculated from the multiple measurements discussed in the section “Textile structure analysis”. Model prediction is the predicted bite resistance. “Safe” represents a fabric that is predicted to have 100% bite protection predicted by the bite-resistance model and “unsafe” means the fabric is predicted to allow at least 1 mosquito bite. Bioassay results are actual measurements of bite resistance. “Pass” indicates the fabric was at least 95% bite resistant by the in vitro or in vivo bioassay. “Fail” indicates a fabric provided less than 95% bite protection.

## 3. Results and Discussions

### 3.1. Mosquito-Bite-Resistant Textile Model

Figure 1A shows an adult female *Ae. aegypti* probing human skin. Figure 1B is a scanning electron microscopy picture (SEM) of a knitted textile. The yarns used to make the textile consisted of a multitude of filament fibers knitted in an intermeshed loop configuration. In a knitted fabric, the spaces between the filaments form pores (Figure 1C) and together with its thickness determine a fabric’s bite resistance to mosquitoes and its comfort to people. Pore diameter and fabric thickness are critical limiting factors for mosquito proboscis penetration of the skin that also affect the thermophysiological comfort of a textile (Figure 1D). Increasing pore diameter improves fabric breathability and comfort but increases the transmission of skin odorants, increasing mosquito landings and biting. Fabrics containing small pores are less attractive to mosquitoes and more bite resistant but have reduced comfort because of reduced air flow. Increasing fabric thickness improves bite resistance but reduces comfort by increasing thermal insulation. A model to predict bite resistance was developed that informed fabric thickness and pore diameter as they related to the morphometrics of the mosquito’s head, antennae, and proboscis, and the mechanism that mosquitoes use for finding and biting through a textile. The three cases considered are illustrated in Figure 1E. Figure 1F describes our overall strategy for developing bite-resistant garments: (i) developing a predictive model based on mosquito head morphometrics; (ii) model validation using mosquito *in vitro* testing of woven filter fabrics, plastic spacers, and 3D spacer fabrics for bite resistance; (iii) development of knitted fabrics for garment construction using the model; (iv) *in vivo* (arm in cage) mosquito testing for bite resistance of these fabrics; (v) garment construction; and (vi) garment walk-in-cage testing for bite resistance; and (vii) manikin comfort tests of the garments.

Figure S1A shows the size of the proboscis where the stylets of the proboscis interlock forming a feeding tube covered by the labium (Figure S1B). Figure S1C shows the stylets, and Figure S1D is an SEM of the mosquito’s proboscis composed of the labrum, maxillae, mandibles, and hypopharynx. The mechanical process of probing skin was described previously [31,32]. The labrum’s diameter was measured in our work as a key parameter for our bite-resistance model. Preventing labrum contact with the skin prevents blood feeding. Figure 2A–D provide a detailed description of Cases 1–3. In Case 1, the pore diameter of the fabric barrier is smaller than the diameter of the labrum (Figure 2B). In Case 2, the pore size of the fabric barrier is larger than the labrum diameter but smaller than the diameter of the mosquito head (Figure 2C). Thus, fabrics with the proper thickness can prevent the labrum tip from contacting skin. In Case 3, the fabric pore size is larger than the head diameter but is smaller than the size of the head plus antennae (Figure 2D). The ice-green vertical bars are the textile barrier, and the red dotted line the critical combination of pore diameter and thickness of the textile barrier.

The critical geometrical relationships of pore diameter and thickness for each case to prevent blood feeding were defined as follows:

Case 1:

(2) $$t=\frac{x}{2\times tan\;\left(\frac{\alpha }{2}\right)\;},\;when\;0\leq x<D$$

(3) $$\frac{D}{2\times tan\;\left(\frac{\alpha }{2}\right)\;}\leq t\leq L_{proboscis},\;when\;x=D$$

Case 2:

(4) $$t=L_{proboscis}+\frac{D_{head}}{2}\left\{1-cos\left[arcsin\left(\frac{x}{D_{head}}\right)\right]\right\},\;when\;D<x\leq D_{head}$$

Case 3:

(5) $$t=L_{\mathrm{proboscis}}+\frac{D_{\mathrm{head}}}{2}+\tan\left(\beta -90\right)\times \left(x-D_{\mathrm{head}}\right),when\;D_{\mathrm{head}}<x\leq L_{\mathrm{antenna}}$$

where *t* and *x* are the thickness and pore diameter of the mechanical barrier, respectively; $L_{\mathrm{proboscis}}$ is the maximum proboscis length; *D* is the maximum diameter of the proboscis tip; *α* is the angle of insertion of the proboscis tip; and *β* is the angle between the antenna and proboscis.

The red dotted lines in Figure 2B–D show the limit between a textile being predicted as unsafe (biting is possible) and safe (biting cannot occur) for Cases 1–3 (for critical combinations of pore sizes and thicknesses as specified by the model). For the model to be feasible, we made the following assumptions: (1) the fabric barrier and proboscis tip were not deformable; and (2) only thickness and pore diameter were considered as structural parameters for the fabric barrier. Figure 2F,G show the correlation between the bite-resistance performance predicted by the model and fabric pore size and thickness, in which the abbreviations of all dimensional values are described in Figure 2E. In Figure 2F,G, the brown dotted lines mark the dimensions of the key factors of the mosquito anatomy, including the head diameter, labrum and its tip length, and diameter and antenna angle from the head and length. The red solid lines are the critical combinations of the fabric pore diameter and thicknesses relative to the mosquito morphometrics that would produce a safe (100% bite resistance shown in green) or unsafe (pink) fabric as predicted by the model.

### 3.2. Mosquito Morphometrics Used to Predict Safe Fabrics

The head diameter (*D*_head_), antenna length (*L*_antenna_), proboscis length (*L*_proboscis_), maximum labrum diameter (*D*), labrum tip length (*L*_tip_) and the tip angle (α) of *Ae. aegypti* adult females are shown in Figure 3A. Each body part was measured from twenty insects. The average values were input into our model to define the fabric thickness and pore diameter and the limit between safe and not safe (Figure 3B). We focused on these limits and produced a variety of barriers of different pore sizes and thicknesses for the experiments (Figure S5A–F) to test the model using our *in vitro* bioassay (Figure S2A). In some cases, these barriers (description follows) were not practical for garment construction but were used because they were optimum for model validation, as explained in the Materials and Methods.

For Case 1, single-filament (woven) filter fabrics (shown in Figure 1Fii and Figure S2B) with different pore sizes and a fixed thickness (Figure S5A,B) were tested using the *in vitro* mosquito-bite-resistance bioassay (Figure 1Fii and Figure S2A). These are technically fabrics, but they are highly resistant to stretch, uncomfortable to wear, and too costly for garment construction. However, they were used for model validation because they were available in precise, different pore diameters and fabric thickness. Highly precision-machined, polypropylene plastic plates (Figure 1Fii and Figure S2C) were used with different pore sizes and thicknesses (Figure S5C,D) to evaluate the model for Cases 2 and 3 using the *in vitro* bioassay. Then, two knit fabrics for Case 1 and two knitted spacer fabrics (shown in Figure 1Fii) for Cases 2 and 3 each with different pore diameters and fabric thickness (Figure S5E,F) were constructed to inform further on Cases 1–3, to better approximate a practical garment application than filter fabrics and plastic plates.

The number of landings and percentage blood feeding for the barriers tested are shown in Figure 4 for our model validation research. Table 1 (group = materials for model validation) relates thickness and pore diameter to the model prediction and whether the barrier failed or passed in preventing mosquito blood feeding. In these experiments, a percentage of blood feeding greater than 5% (bite resistance was lower than 95%) was considered a failure for the barrier in preventing blood feeding. In Figure 3B, the left and right graphs relate the pore size and thickness for the filter fabrics and plastic plates, respectively, with the model prediction of what would be safe and unsafe. Only one (plastic plate S7, Table 1) out of the 18 barrier materials tested (filter fabrics, plastic plates and knit fabrics) failed to provide bite protection when the model informed the barrier should be safe. This failure in the model corresponds to the red dot in the green area in Figure 3B, the right graph. Those barriers (green color dots) located in the safe area exhibited bite resistance against mosquitoes of at least 95%, as the model predicted for the filter fabrics and plastic plates. The model was 100% accurate in predicting safe and unsafe for both the knit and knitted spacer fabrics (Table 1, T1–T6).

These results suggest that the model we developed was reliable for predicting mosquito-bite resistance against the lab-reared mosquito, *Ae. aegypti*, and was 100% reliable in our studies of the knits and spacer knits tested. Additional testing will be needed in the future, to determine if our model translates to other mosquito species and to mosquitoes in the field. Regarding for the economy of time and resources, we argue concentrating on one species was a reasonable approach for our studies and proof of concept.

### 3.3. Finite Element Analysis

In our validation studies, a barrier was considered safe when bite resistance was 95% or higher. When pore sizes and thickness approached the limit between safe and unsafe (Figure 3B left graph for filter fabrics and right graph for plastic plates), some blood feeding occurred at a low percentage, 5% or less (Figure 4A–D). This was also the case for the knits tested (Figure 4E,F). There are two possible reasons. First, the labrum diameter of some mosquitoes may have been smaller than the average value (27.5 µm) used in the model, allowing some mosquitoes to penetrate the barriers. Second, the barrier may have deformed under the pressure of the proboscis and enlarged the pores causing failures. In the latter case, this would not be an issue with the plastic plates but could be a factor for the textiles tested.

To investigate the interaction between proboscis and textile structure, the elastic modulus and geometry of the labrum were measured to establish a finite element labrum model. Figure 3C shows the anatomy of the proboscis tip. Figure 3D is the nanoindentation curve for the labrum, which was used to obtain the elastic modulus for the property parameters needed for the model. The woven (filter) fabric used in our validation studies (Section 3.2), W1 to W4 (Table 1), were modeled to better understand how the labrum might deform textiles in general. Figure 3E illustrates the four patterns. Figure 3F shows one example of the penetration model for the labrum on the W2 woven fabric, and Figure 3G shows the time course of penetration. For W1, the labrum interaction with the textile is less since the labrum can easily go through the fabric. However, W3 and W4 in Figure 3E are more dense structures with the pore size below that of the labrum diameter, not allowing free labrum penetration through the pore. Therefore, W2 with a pore diameter of 18 um was selected to show fabric deformation subjected to labrum penetration. It was found in our research that the labrum can move the filament yarn and push through the W2 filter fabric over time (Figure 3G) for a blood meal. This is the reason that W2 located near the boundary line failed in resisting some mosquito bites. Figure 3L shows the change curves for the pore diameters of each woven structure. After labrum penetration, W1 and W2 were enlarged more than the labrum diameter and therefore would fail in preventing blood feeding because the structures were deformed. Although pores on W3 and W4 demonstrated deformation, the pore diameter was still below the labrum diameter, which enabled the structure to prevent blood feeding. In summary, in addition to the importance of pore size and thickness, the finite element analysis informs that micromechanical deformation of the fabric in response to the pressure exerted by the proboscis pushing-through the fabric can affect blood-feeding success. Yarn chemistry and methods of weaving and knitting will impact deformation and, therefore, bite resistance. It would also be expected that variation in labrum diameter in the mosquito population will have an impact.

### 3.4. Development of Fabrics for Garment Construction

Once the model was validated for Cases 1–3, textiles were developed for the construction of a garment for final proof of concept that non-insecticide clothing could be bite resistant to mosquitoes and also comfortable. For these studies, bite resistance was measured with arm-in-cage bioassays (Figure S3A) with a textile considered safe if the bite resistance was 95% or higher. For Case 1, the knitted fabrics were H and B (Table 1) and shown in Figure S3B,C, respectively, and in Figure 1Fiii. For Case 2, the knitted spacer fabric was S (Table 1) and shown in Figure S3D, front and back, and Figure 1Fiii. Thickness and pore diameters are shown in Figure S5G,H, respectively, and the model prediction and bioassay results are in Table 1. The model was correct in all cases (see group = fabrics used in garments) in successfully predicting bite resistance. Accordingly, these textiles were used for garment construction.

### 3.5. Bite Resistance of an Insecticide-Treated versus Non-Insecticidal Textile

Permethrin-treated textiles are a widely used technology to prevent mosquitoes from biting people. Permethrin exhibits mosquito contact toxicity but also spatial repellency. Figure 3I shows the number of landings on fabric P (a permethrin-treated commercial fabric; detail on pore size and thickness in Table 1), which was lower (*p* < 0.01) than that for fabric H, the non-insecticidal superfine knit. Fabric P demonstrated spatial repellency presumably because of permethrin in the cloth whereas fabric H did not. Fabric H had a higher number of landings because mosquitoes were not repelled and landed on the fabric repeatedly in attempts to find a suitable location to penetrate the fabric. High landings without bites indicated the fabric structure has breathability but with pores sufficiently small for high bite resistance. Figure 3J shows that the percentage of blood-fed mosquitoes in the arm-in-cage studies for fabric P was three times higher than fabric H (*p* < 0.05). Although fewer mosquitoes landed on fabric P, a larger percentage of the mosquitoes that landed were able to penetrate the fabric and obtain a blood meal. In contrast, fabric H with smaller pore diameters and no insecticides resisted mosquito bites at a higher level.

These studies demonstrated that high bite resistance across a textile can be achieved that far succeed one commercial permethrin-treated fabric under high biting pressures in an arm-in-cage bioassay. Higher landings with no spatial repellency on the insecticide-free cloth would be expected to reduce biting on uncovered skin, especially when the proportion of uncovered to covered skin is small; in this case, the mosquitoes are probing the cloth and not being pushed to unprotected skin. However, more detailed studies are needed to address how an insecticide-treated textile versus a non-insecticide-treated textile, such as fabric H, would protect uncovered areas of the body.

### 3.6. Comfort and Bioassay Evaluation of Prototype Garments

The final step in demonstrating the proof of concept that insecticide free textiles can be used to protect humans from mosquito blood feeding and at the same time be comfortable, was to construct garments with the knits that our model predicted would be safe (fabrics H, B, and S, Table 1). These fabrics were used to construct a protective undergarment (a base layer garment; NCSU base layer, Table 1, and shown in Figure S6A and Figure 1Fv) and shirt (NCSU combat shirt, Table 1 and shown in Figure S6B and Figure 1Fv). These garments were tested in walk-in-cage bioassays to evaluate the mosquito-bite resistance where the threshold for success was no bites. A sweating manikin test was conducted to create whole-body heat loss maps for fabrics in different body zones to understand the heat and moisture resistance properties of our mosquito-bite resistant garments compared to commercially available garments.

Garments were tested for heat loss using a sweating manikin (Figure 5A). The garments included I, an Under Armour base layer; II, the NC State base layer developed using our model; III, a US army-issued combat shirt (provided by the US DOD); and IV, the NC.

State-developed, next-generation combat shirt, using our model. The same style of garments had similar heat-loss maps (Figure 5A), which indicated equivalent levels of thermal management. In the maps for garments III and IV, the blue color of IV is darker than III due to an innovative design that incorporated a 3D spacer fabric (Figures S3D and S6B and Figure 1Fiii) predicted to be bite resistant by our model but with open pores into the chest and arms area for heat management (Figure 5A).

The insulation values for both of our developed garments (Figure 5B) were smaller than their counterparts of the same style. This finding indicated that the NC State base layer and the NC State combat shirt had favorable thermal exchange as well as minimal heat accumulation, making the garments more comfortable to wear in warm weather. The Predicted Heat Loss Potential (*Q*_predicted_, W/m^2^) is a projection of the total amount of heat that could be transferred from the manikin to the ambient environment for a given condition, which was calculated using thermal and evaporative resistance values (see details in Table S1). In this case, the *Q*_predicted_ of garments II (NC State base layer) and IV (NC State combat shirt) exhibited higher values than their counterparts (Figure 5C), which indicated they possessed superior comfort performance in both thermal and moisture management.

The NC State base layer and the NC State combat shirt were tested in walk in cage bioassays under heavy mosquito biting pressure with the human subject standing and sitting for 10 min in each posture (Figure 5D,E). The NC State combat shirt provided 100% protection against mosquito bites. However, the human subject wearing the NC State base layer received bites on the back and shoulders and the level of overall average protection was 96.5% (7 bites per 200 mosquitoes). When the base layer is used as an undergarment under a uniform, protection would be 100% (data not shown). This result on biting in the test reported was attributed to deformation of the knitted fabric on the shoulders where the fabric stretched, increasing the pore diameter of the fabric. We measured the fabric length during standing and sitting. Fabric H was estimated to have a 9.47% increase in stretch from the standing to the sitting postures. We conducted a virtual tensile experiment using an FEA model to investigate the change in the pore diameter of fabric H (see details in Figure S7). The tensile behavior of the fabric showed a directionality of stretch in which the wale direction exhibited a smaller deformation compared with the course direction, as shown in Figure S7C. The pore diameter also exhibited directional deformations in the course and wale directions, as shown in Figure S7D,E. In order to improve the bite resistance, a double layer of fabric H was stacked on the shoulder area (yoke), which partially covered the back of the human subject. The stacked orientation for both layers were perpendicularly aligned with each other, which reduced the fabric deformation during sitting and movement; this treatment also misaligned the pores of both fabrics. This improved the garment’s bite resistance and provided 100% bite protection in walk-in-cage bioassays. Our two final garments listed in Table 1 were 100% bite proof in walk-in-cage tests. Notably, when the base layer was used as an undergarment under a uniform, protection was 100% (data not shown). In summary, preventing human–vector contact is an effective way to protect people from mosquito bites as well as to eliminate the threat of mosquito-borne diseases. We developed a mathematical model to predict the bite resistance of non-insecticidal textile barriers. Our model was verified through in vitro bioassays, using woven fabrics, plastic spacer plates, and knitted and knitted spacer fabrics, which showed that the model could accurately predict the bite resistance of mechanical barriers. The model was then used to develop comfortable and wearable textiles for garments. When compared with a permethrin-treated fabric, our fabrics development for garments had a higher bite resistance with a predicted higher level of protection for exposed skin; however, the latter needs further study. Then, the prototype garments were constructed with these textiles. These garments exhibited superior comfort performance compared to similar commercial garments and 100% mosquito-bite resistance. Use of our model in the future will facilitate development of other, highly effective and comfortable bite-resistant fabrics solely based on textile structure without the need for an insecticidal treatment to prevent mosquito biting, and thus can be used to produce mosquito-bite-proof clothing for everyday use.

## 4. Conclusions

Preventing human-vector contact is an effective way to protect people from mosquito bites as well as to eliminate the threat of mosquito-borne diseases. We developed a mathematical model to predict the bite-resistance of non-insecticidal textile barriers. Our model was verified through *in vitro* bioassays, using woven fabrics, plastic spacer plates and knitted and knitted spacer fabrics, which showed that the model could accurately predict bite-resistance of mechanical barriers. The model was then used to develop comfortable and wearable textiles for garments. When compared with permethrin-treated fabric, our fabrics development for garments had a higher bite-resistance with a predicted higher level of protection for exposed skin; the latter needs further study however. Then prototype garments were constructed with these textiles. These garments exhibited superior comfort performance compared to similar commercial garments and 100% mosquito bite-resistance. Use of our model in the future will facilitate development of other, highly effective and comfortable bite resistant fabrics solely based on textile structure without the need for an insecticidal treatment to prevent mosquito biting and can be used to produce mosquito bite proof clothing for everyday use.

## Figures and Tables

**Table 1:** Barrier materials studied, their abbreviation, measured thickness and pore diameter, model prediction, and bite-resistance bioassay results.

Group	Type	Name	Abbreviation	Thickness (mm), Pore Diameter (µm)	Model Prediction ^†^	Bioassay Result ^††^
Materials for model validation (test, in vitro)	Stable structures	Case 1 woven filtration fabrics	W1	0.052, 25	unsafe	fail
			W2	0.040, 18	unsafe	fail
			W3	0.058, 16	safe	pass
			W4	0.082, 8	safe	pass
		Case 2 plastic plates	S1	2.1, 500	unsafe	fail
			S2	2.1, 800	unsafe	pass
			S3	2.5, 500	safe	pass
			S4	2.5, 800	unsafe	fail
			S5	2.5, 1250	unsafe	fail
		Case 3 plastic plates	S6	2.72, 800	safe	pass
			S7	2.75, 1250	safe	fail
			S8	3, 1250	safe	pass
	Textile materials	Case 1 fabrics	T1	0.29, 36	unsafe	fail
			T2	0.26, 16	safe	pass
		Case 2 spacer fabrics	T3	2, 120	unsafe	fail
			T4	3.2, 420	safe	pass
		Case 3 spacer fabrics	T5	2, 940	unsafe	fail
			T6	3, 770	safe	pass
Fabrics used in garments (test, in vivo)	Textile materials	Case 1 fabric H	H	0.3, 28	safe	pass
		Case 1 fabric B	B	0.68, 0	safe	pass
		Case 2 spacer fabric S	S	2.48, 420	safe	pass
Permethrin-treated fabric (test, in vivo)	Chemical-treated textile materials	InsectShield^®^ T-shirt fabric	P	0.61, 90	unsafe	fail
Garments (test, walk-in cage)	Garments	Under Armour^®^ men’s base 1.0 crew	I	--	--	--
		NCSU base layer	II	--	--	pass
		Winter army combat shirt	III	--	--	--
		NCSU shirt	IV	--	--	pass

Note: ^†^ Model prediction means the bite resistance of each fabric predicted by the bite-resistance model. “Safe” means the fabric has 100% bite protection and “unsafe” means the fabric is predicted to allow mosquito biting (based on our bite-resistance model). ^††^ Bioassay result is an actual measurement of bite resistance. For in vitro and in vivo tests, “Pass” means the fabric demonstrated at least 95% bite protection. For the walk-in-cage test, pass means no bites.

## Data Availability

All data are available in the main text or the supplementary materials.

## Supplementary Materials

The following are available online at https://www.mdpi.com/article/10.3390/insects12070636/s1. enclosed in attachment.
