# Supplementary material for: Mosquito-Textile Physics: A Mathematical Roadmap to Insecticide-Free, Bite-Proof Clothing for Everyday Life
Source: Insects. 2021 Jul 13;12(7):636. doi: 10.3390/insects12070636 (PMC8306269; doi:10.3390/insects12070636)
Supplement: Supplementary file 1 [file insects-12-00636-s001.zip › insects-1281503-supplementary.pdf]

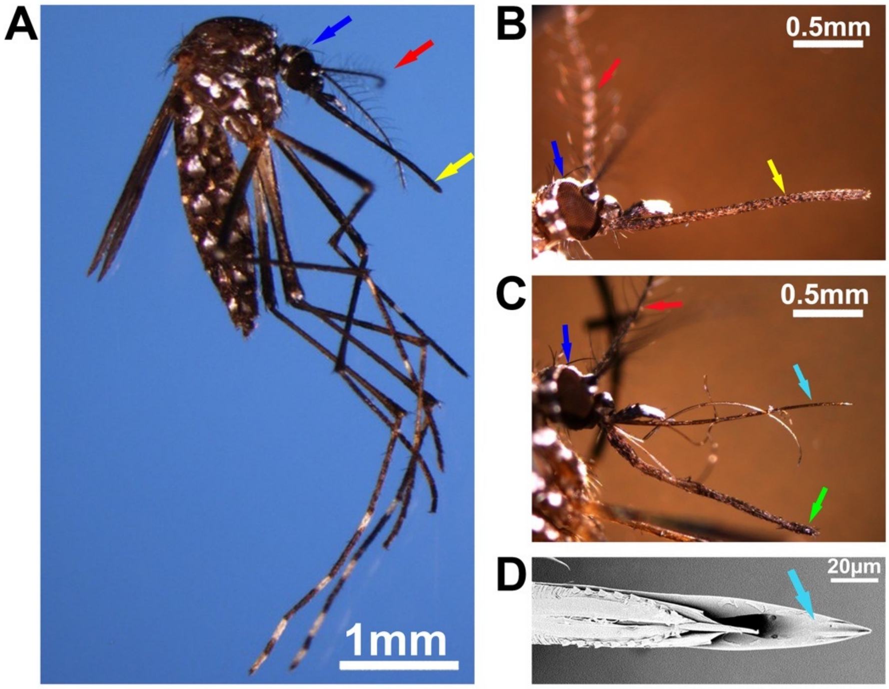

Figure S1. Morphometrics of the adult, female yellow fever mosquito, *Aedes aegypti*. (**A**) Microscope image of *Ae. aegypti* adult female. (**B**) Zoomed image of mosquito head (blue arrow), antenna (red arrow) and proboscis (yellow arrow), of which geometries were used to develop Case 2 and Case 3. (**C**) labrum (ice blue arrow) and labium (green arrow). (**D**) SEM image of the labrum tip of the proboscis (ice blue arrow). The geometry was used to develop the Case 1 model.

.

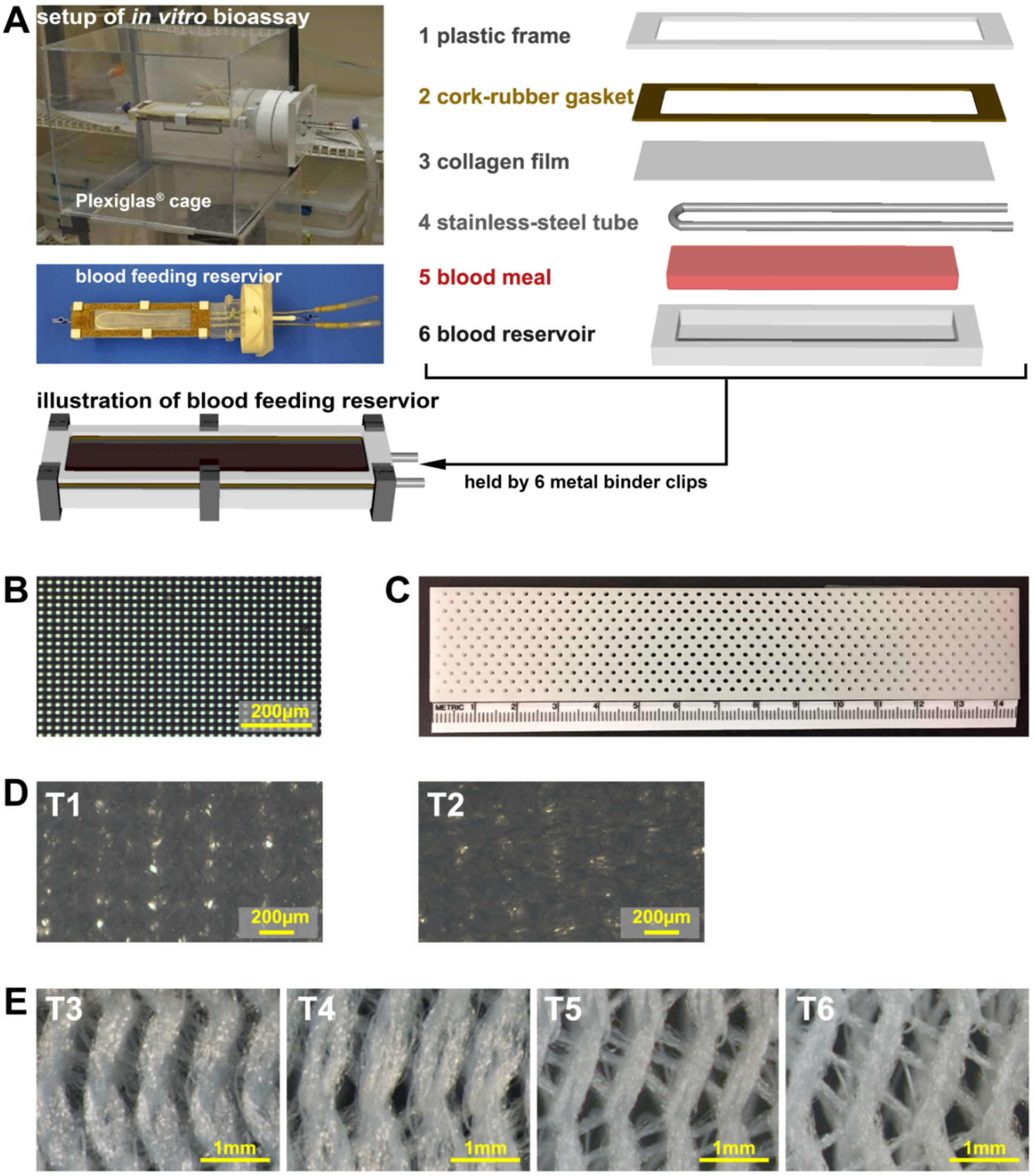

Figure S2. *In vitro* bioassay architecture and materials for experimental validation of the predictive model. (**A**) *In vitro* bioassay setup. Blood feeding reservoir (BFR) and BFR inserted into mosquito bioassay cage. See Materials and Methods for detailed description. Stable structures used for validation were (**B**) Woven barriers for validating Case 1, (**C**) Plastic plates with bored holes for Case 2 and Case 3, (**D**) Knitted textiles T1-T2 for Case 1, and (**E**) 3-D spacer knitted fabrics T3-T4 for Case 2 and T5-T6 for validating Case 3 (see Fig. 1 for description of Cases and fig. S5 and Table 1 for pores sizes and thickness).

.

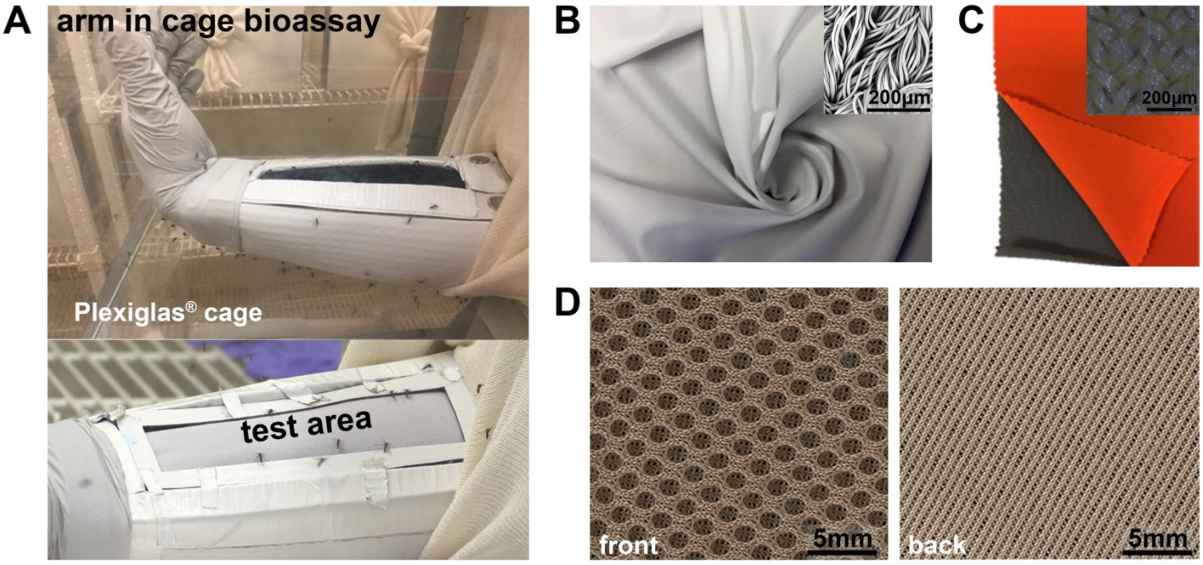

Figure S3. *In vivo* bioassay architecture and textiles for experimental validation of the predictive model. (**A**) *In vivo* (arm in cage bioassay) with human arm covered with fabric and protective sleeve. The predicted safe fabrics used to make garments include: (**B**) Case 1 H fabric which is an ultra-fine thin synthetic polymeric knit with small pores (pore diameter ~28 μm that can block labrum penetration verified by the arm in cage bioassay); (**C**) Case 1 B fabric composed of two layers of the H fabric; and (**D**) the Case 2 S fabric which is a 3-D warp knit textile with high thickness. The purpose of using the S fabric is because the dense back layer can absorb sweat, while the front openings allows air flow to bring water vapor away from the back layer to the outside and to accelerate heat exchange. The structure would bring a cool feeling to the garment. Before these are made into garments, the mosquito bite resistance of these fabrics was evaluated using the arm in cage bioassay. (see fig. S5 and Table 1 for pore sizes and thickness).

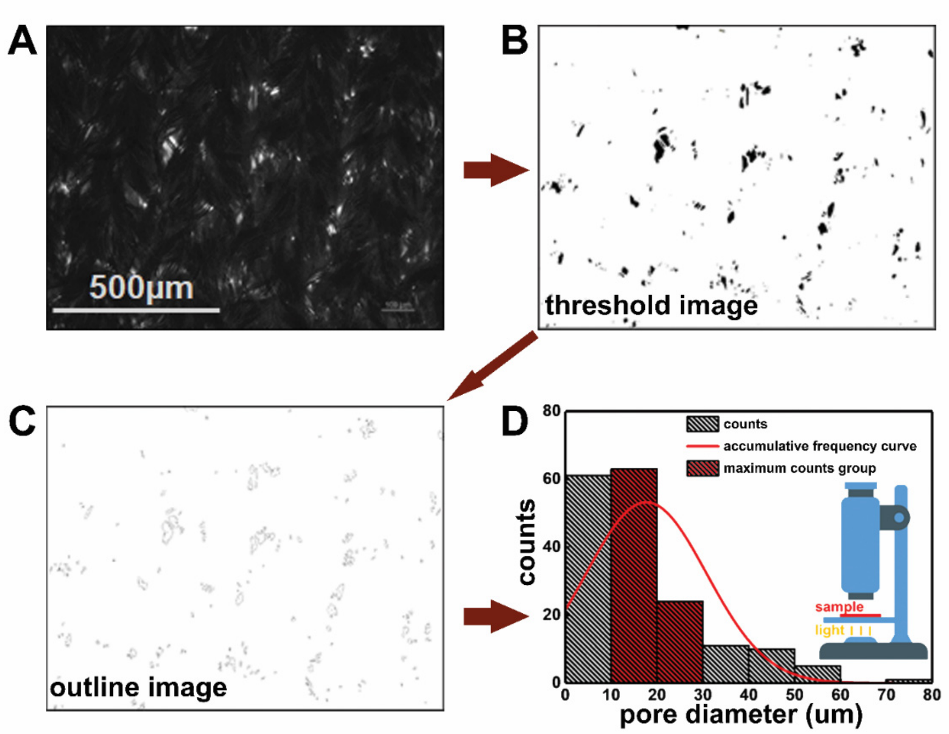

Figure S4. Measurement of knit fabric pore diameter by digital microscopy using ImageJ software. (**A**) Digital microscope image from a Bausch & Lamp Monozoom-7 Zoom Microscope (Bridgewater, NJ, USA). During image capture, white light illuminates the cloth from underneath from the built-in source of illumination for the microscope. This allows a determination of pore distribution across the textile from this transmitted light. (**B**) The image from the microscope was imported into ImageJ and processed into a black/white threshold (binary) image. (**C**) The pore edges (image outline) were found to accurately define the pore shapes that could be used for obtaining the Feret's diameter and pore diameter distribution. (**D**) Pore diameter distribution and fitted curve to determine the peak distribution range of pore diameter for 12 maximum diameters as well as an illustration of the digital microscope. See Materials and Methods for more detail.

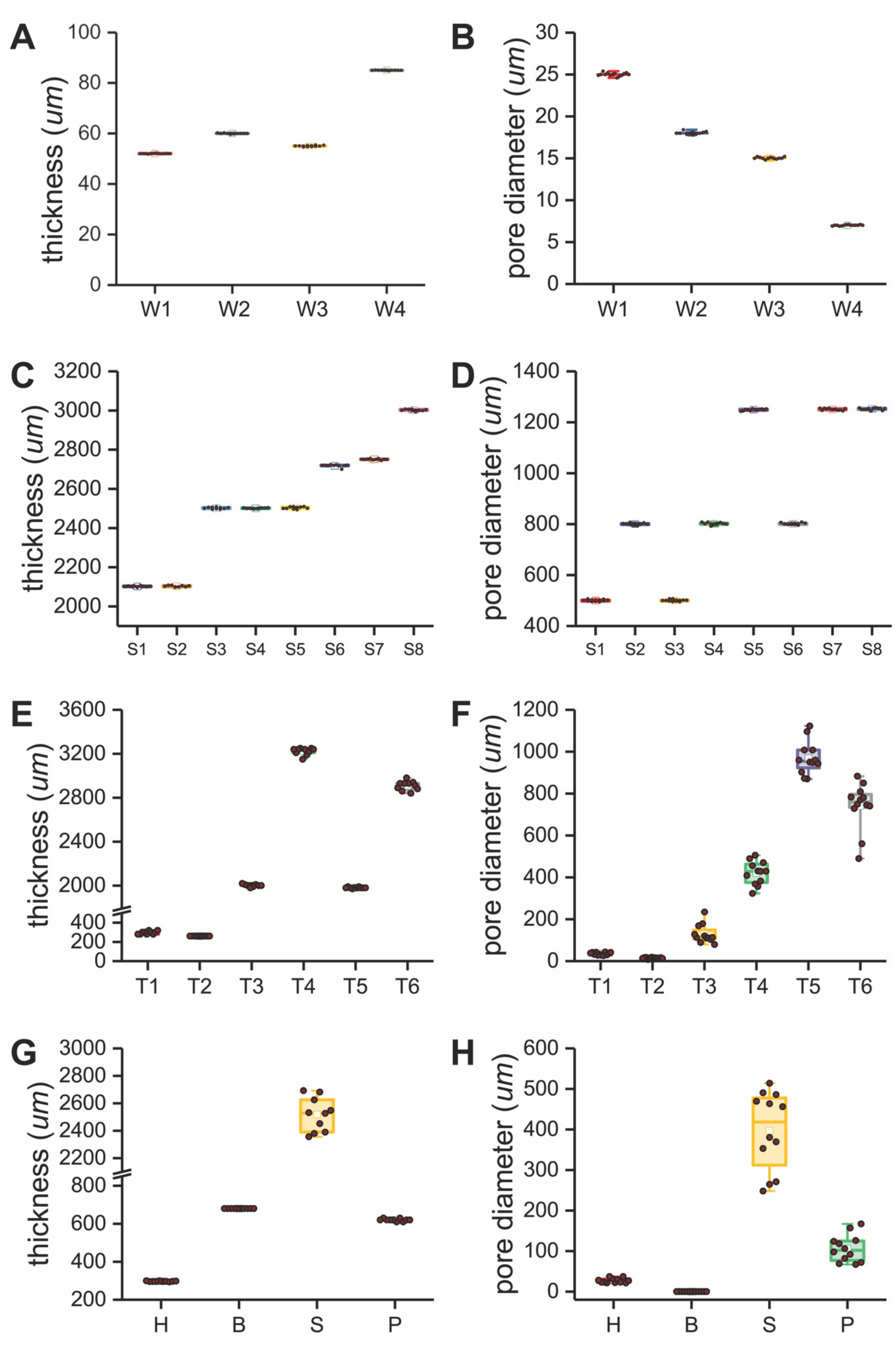
A
thickness (um)
W1
W2
W3
W4
B
pore diameter (um)
C
thickness (um)
S1
S2
S3
S4
S5
S6
S7
S8
D
pore diameter (um)
E
thickness (um)
T1
T2
T3
T4
T5
T6
F
pore diameter (um)
G
thickness (um)
H
B
S
P
H
pore diameter (um)

Figure S5. Construction parameters for materials and fabrics used to validate our resistance model and for assembly of protective garment. (**A,B**) Thickness and pore diameter of Case 1 woven structures (W = Woven structures, W1 to W4)(fig. S2 B). (**C,D**) Thickness and pore diameter of Case 2 and 3 plastic plates (S = plastic spacer, S1 to S8)(fig. S2 C). (**E,F**) Thickness and pore diameter of fabrics T1 to T6 used for model validation (T = Textile materials, T1 to T6) (Figure S2 D,E). (**G,H**) Thickness and pore diameter of candidate fabrics H, B and S for making garments (Figure S3 B–D) and a comparison with a permethrin-treated fabric P (H = high density fabric, B = bonded fabric, S = 3-D spacer fabric, P = permethrin treated fabric). (see Fig. 1 for explanation of Cases).

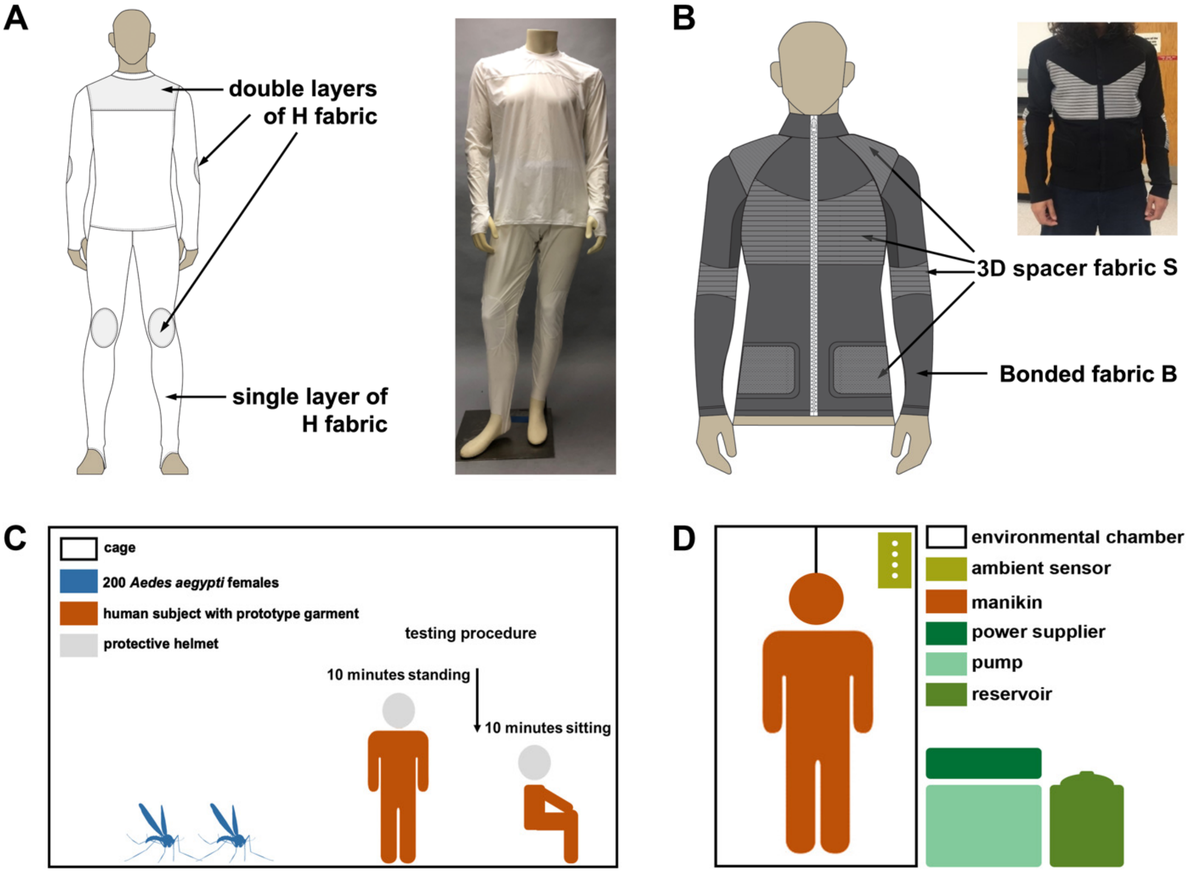

Figure S6. NCSU garments, *in vivo* walk-in-cage study and comfort testing. (**A**) Design illustration and photograph of the NCSU base layer. (**B**) Design illustration and photograph of the NCSU shirt. (**C**) Schematic of walk-in-cage bioassay (see Materials and Methods for more detail); and (**D**) schematic of sweating manikin test for comfort evaluation of the prototype garments. The manikin surface is composed of 34 separate sections, each of which were equipped with its own sweating, heating and temperature measuring system. There were 139 "sweat glands" that distributed moisture on a custom made body suit that acted as "manikin skin". The manikin surface temperature was continuously controlled on each section of the manikin with analog signal inputs from a Resistance Temperature Detector (RTD). RTDs were distributed over an entire section embedded below the "manikin skin" and conducted measurements in a form that all areas were equally weighted and provided an average temperature for each section. The manikin was housed in a climate-controlled chamber (Fig. 5A, top row of pictures). Preheated water was supplied from a reservoir located outside of the environmental chamber to produce sweat. An internal sweat control system distributed moisture to the 139 "sweat glands". Each sweat gland was individually calibrated and the calibration values were used by control software to maintain the sweat rate of each body section. See Materials and Methods for more detail.

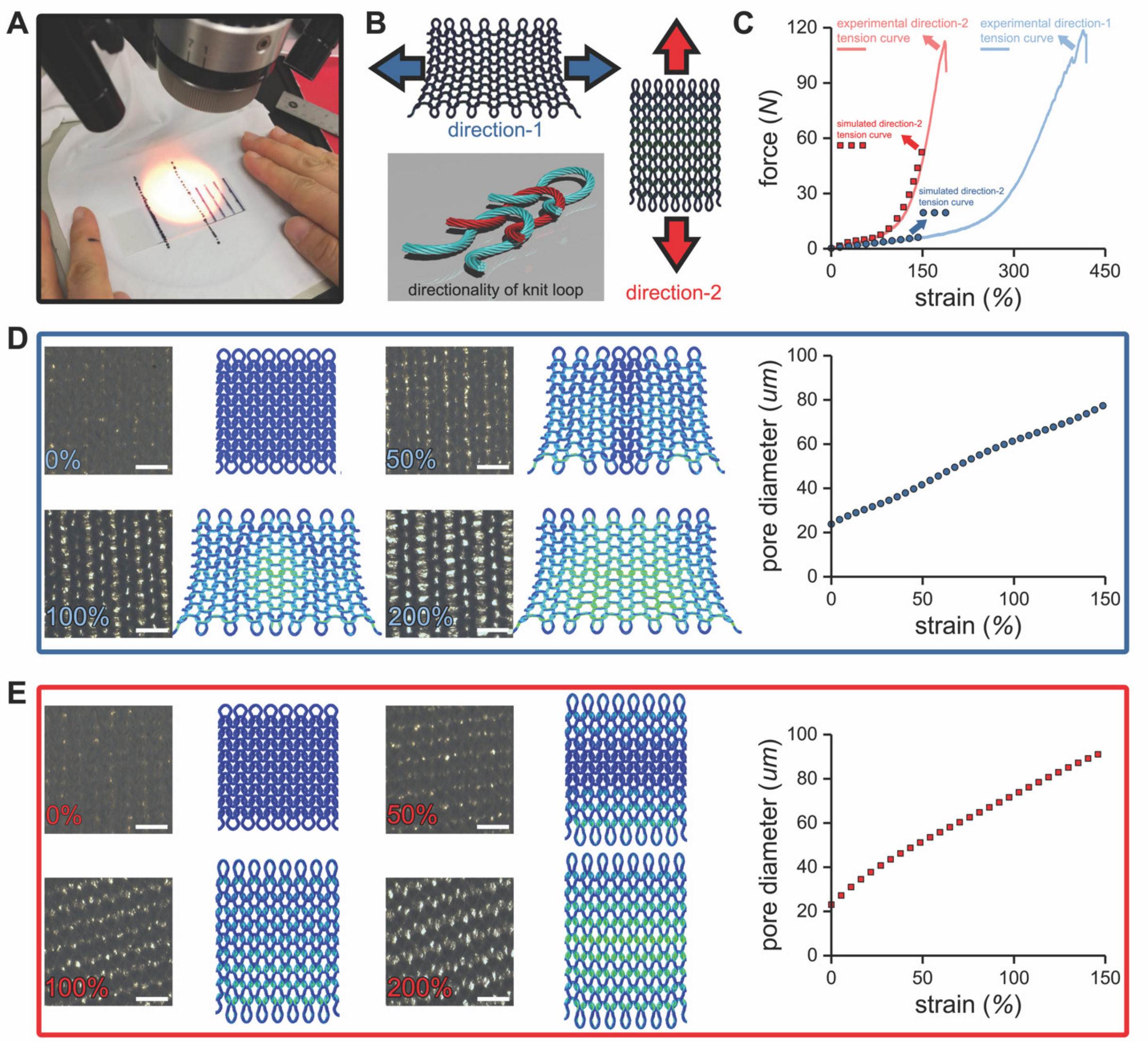

Figure S7. Pore diameter measurements of stretched fabric. (**A**) A microscope was used to capture an image of the pore distribution of the manually stretched fabric H. Prior to image capture, fabric H was marked with two reference lines. We covered a transparent glass marked with predefined distance on top of the H fabric. Then by hand we stretched the fabric to each line marked on the glass plate followed by obtaining an image of the change in pore size and distribution. These measurements were used to compare with our simulations in D and E. (**B**) Illustration of the direction that tension was applied and the corresponding directionality of stretch of the knit loop. The knit loop had an unbalanced structure that altered the directional mechanical property for the fabric. (**C**) Force-strain curves of the experimental tension and simulated tension. The simulated curves were only extended to 150% stretch because this endpoint represented an extreme stretched scenario. Deformation in the course direction (direction-1) showed a higher extensionality than the wale direction (direction-2). (**D**) Pore deformation under the course direction tension. (E) Pore deformation under the wale direction tension. Scale bars in (D) and (E) = 500µm.

Table S1. Sweating manikin test results (raw data, I_t and $Q_{predicted}$ were analyzed by Student's T-test in Fig. 4, B and C).

Garment	Repetition	R_t	R_{et}	R_{cl}	R_{ecl}	I_t	i_m	$Q_{predicted}$
I *Under Armour base layer*	1	0.1221	0.01515	0.0669	0.00681	0.79	0.49	317.2
	2	0.1206	0.01542	0.0654	0.00708	0.78	0.48	313.1
	3	0.1243	0.01547	0.0691	0.00713	0.80	0.49	312.2
II *NCSU base layer*	1	0.1107	0.01510	0.0560	0.00673	0.71	0.45	325.5
	2	0.1127	0.01439	0.0580	0.00601	0.73	0.48	337.2
	3	0.1126	0.01516	0.0579	0.00679	0.73	0.45	324.5
III *Military shirt*	1	0.2015	0.03062	0.1460	0.02148	1.30	0.40	165.7
	2	0.2049	0.03139	0.1494	0.02225	1.32	0.40	162.8
	3	0.2030	0.02893	0.1475	0.01979	1.31	0.43	172.5
IV *NCSU shirt*	1	0.1518	0.02022	0.0964	0.01084	0.98	0.46	239.8
	2	0.1584	0.02001	0.1030	0.01064	1.02	0.48	241.6
	3	0.1632	0.01989	0.1077	0.01052	1.05	0.50	242.7
